# Intensity-based hierarchical Bayes method improves testing for differentially expressed genes in microarray experiments

**DOI:** 10.1186/1471-2105-7-538

**Published:** 2006-12-19

**Authors:** Maureen A Sartor, Craig R Tomlinson, Scott C Wesselkamper, Siva Sivaganesan, George D Leikauf, Mario Medvedovic

**Affiliations:** 1 Department of Environmental Health, University of Cincinnati, Cincinnati, OH, USA; 2 Center of Environmental Genetics, University of Cincinnati, Cincinnati, OH, USA; 3 Dartmouth College, Departments of Medicine and Pharmacology & Toxicology, Dartmouth Hitchcock Medical Center, Lebanon, NH, USA; 4 Mathematical Sciences Department, University of Cincinnati, Cincinnati, OH, USA; 5 Biomedical Informatics Division, Cincinnati Children's Hospital Medical Center, OH, USA

## Abstract

**Background:**

The small sample sizes often used for microarray experiments result in poor estimates of variance if each gene is considered independently. Yet accurately estimating variability of gene expression measurements in microarray experiments is essential for correctly identifying differentially expressed genes. Several recently developed methods for testing differential expression of genes utilize hierarchical Bayesian models to "pool" information from multiple genes. We have developed a statistical testing procedure that further improves upon current methods by incorporating the well-documented relationship between the absolute gene expression level and the variance of gene expression measurements into the general empirical Bayes framework.

**Results:**

We present a novel Bayesian moderated-T, which we show to perform favorably in simulations, with two real, dual-channel microarray experiments and in two controlled single-channel experiments. In simulations, the new method achieved greater power while correctly estimating the true proportion of false positives, and in the analysis of two publicly-available "spike-in" experiments, the new method performed favorably compared to all tested alternatives. We also applied our method to two experimental datasets and discuss the additional biological insights as revealed by our method in contrast to the others. The R-source code for implementing our algorithm is freely available at .

**Conclusion:**

We use a Bayesian hierarchical normal model to define a novel Intensity-Based Moderated T-statistic (IBMT). The method is completely data-dependent using empirical Bayes philosophy to estimate hyperparameters, and thus does not require specification of any free parameters. IBMT has the strength of balancing two important factors in the analysis of microarray data: the degree of independence of variances relative to the degree of identity (i.e. *t*-tests vs. equal variance assumption), and the relationship between variance and signal intensity. When this variance-intensity relationship is weak or does not exist, IBMT reduces to a previously described moderated t-statistic. Furthermore, our method may be directly applied to any array platform and experimental design. Together, these properties show IBMT to be a valuable option in the analysis of virtually any microarray experiment.

## Background

Identifying differentially expressed gene transcripts is the most common task in analyzing microarray data. The current state-of-the-art in microarray design and analysis involves identifying differentially expressed genes by assessing the statistical significance of observed ratios in replicated microarray hybridizations with independent samples [1]. After performing the initial data processing designed to remove several important sources of variation, the traditional and most commonly used approach is to treat each probe (or probe set in the case of Affymetrix GeneChips) as an independent experiment. After performing usual statistical analysis such as the *t*-test or analysis of variance, individual *p*-values are adjusted for the number of hypotheses performed [2].

Considering data for each probe/gene transcript separately when testing for differential expression is statistically inefficient. The estimates of variance are often poor due to small sample sizes. However, additional information may be gained by combining variance estimates across all genes, and methods that exploit this information improve results [3-9]. Several of these methods use hierarchical Bayesian models or other methods for calculating "moderated" variances for individual genes, weighted averages of the gene-specific sample variances and the pooled estimate of variance calculated from all genes [3,4,6,10-12]. Empirical comparisons of such procedures have demonstrated that the gain in statistical power can be substantial [10]. Others use more heuristic types of arguments to modify artificially small variance estimates that are likely a consequence of random fluctuations in the data [9,13].

An additional source of information not commonly utilized in the statistical analysis of microarray data is the well documented dependence of gene variances on overall expression level of corresponding genes [3,11,14,15]. One notable exception is Cyber-T [3], a hierarchical Bayesian method in which gene-specific "prior" variances are calculated within a window of genes with similar expression levels. Interestingly, Cyber-T performed best in the analysis of a "spike-in" Affymetrix experiment [14]. However, the applicability of Cyber-T is somewhat limited in that two important parameters, the window size and the prior degrees of freedom, need to be specified by users, and it supports only t-tests, paired t-tests, and one-way Analysis of Variance (ANOVA). In contrast to Cyber-T, the moderated-T procedure proposed by Smyth [12] (SMT), and implemented in the *eBayes *function in the *limma *package of Bioconductor, uses an empirical Bayes framework to estimate all parameters from data and it can be used to test any hypothesis within the traditional linear models framework. However, it does not utilize the relationship between variances of expression level measurements and their magnitude.

Recently, Fox and Dimmic proposed an extension of Cyber-T, (Fox), for two-sample comparisons. Like Cyber-T, this method assumes a hierarchical Bayesian model and uses a moving window average to calculate the prior variances. Although they remove some of the *ad hoc *nature of Cyber-T, the window size is still specified by the user, and the prior degrees of freedom are calculated based on the moving window size, by assuming genes with similar expression levels have identical variance. This is an important contrast with Smyth's and our method [12]. Furthermore, Fox's method is limited to simple two-sample comparisons and cannot account for the dye-effect in dual-channel microarrays. Here we describe and evaluate a new Bayes moderated-T statistic which we refer to as IBMT (Intensity-Based Moderated T-statistic). IBMT is an extension of SMT [12] and accounts for the dependence of variance on gene signal intensity. Like SMT, IBMT can be used with any experimental design, including but not limited to experiments with multiple treatments and/or both technical and biological replicates, experiments with a continuous covariate, and dual-channel experiments with dye-effects. It can also be used with any array platform, for example Affymetrix, dual-channel, tiling arrays, etc. Similar to Smyth, we use empirical Bayes (EB) theory to estimate all parameters of the hierarchical Bayesian model. We use non-parametric local regression to functionally relate variance and absolute gene expression measurements. This possibility has been previously proposed but has not been further explored [3].

In this paper, we describe the hierarchical model for gene expression data, detail the procedure for estimating all parameters in the model, and describe the testing procedure for identifying differentially expressed genes. In simulations carefully designed to mimic real microarray data [16-18], we determine that overall our method outperforms all other tested methods, including the simple T-statistic, fold change cut-off, SMT, and Fox. We demonstrate that IBMT performs as well as, or better than any other tested method in when using simulated data and "spike-in" Affymetrix experiments [14]. We also apply our method to two experimental microarray datasets [19] that due to their experimental designs, cannot be correctly analysed with previously proposed methods that account for the variance-intensity relationship (CyberT and Fox). We find that our method generally resulted in higher significance of Gene Ontology (GO) [20] groups when testing for an enrichment of differentially expressed genes. We also provide examples of how our method results in biological conclusions that may not have been attained using an alternative method.

## Results and discussion

### Intensity-based Bayesian model

**Figure 1:**
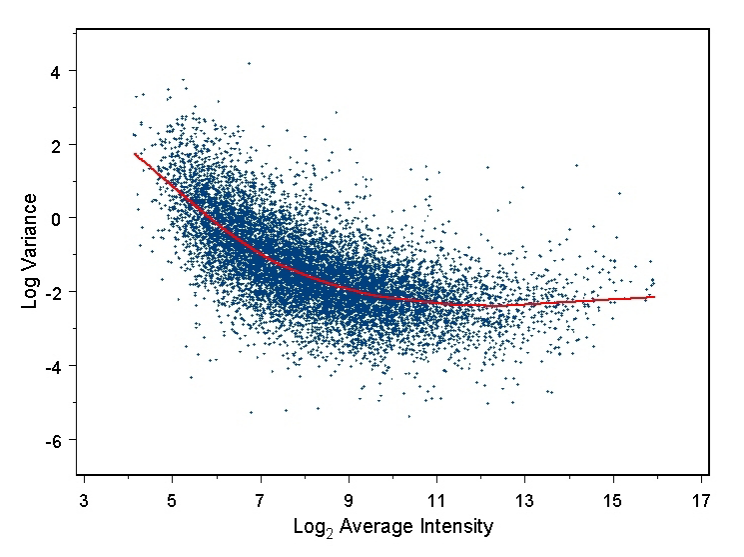
**Dependence of gene variance on average log-intensities**. Typical example of the form of dependency of log-variance on average log-spot intensity. Red line was determined using local regression. Data were from mouse embryo fibroblast *Ahr*^-/- ^dataset.

Figure 1 displays an example of the dependence of gene variance on expression level, taken from the MEF *Ahr*^-/- ^dataset (see Methods section), similar to the observed dependency published previously [3]. The fact that such a dependence exists is intuitive, in view of how the data are measured from the microarray images. Spots with low fluorescence level will likely have fewer pixels measured, and the resulting estimate of expression is an average or median of fewer or lower numbers. Furthermore, transcripts that are lowly expressed are changed by a greater proportion by the addition of a few labeled transcripts, and thus may actually vary more in biological tissue samples. This relationship between variance and expression level can be modeled as

*s*_0*g*_^2^(*α*_*g*_) = *f*(*α*_*g*_) + *ε*_*g *_    (1)

where the average log-expression level of gene *g *is denoted by *α*_*g*_, *f*(*α*_*g*_) is some function of *α*_*g *_defined on the range of *α*_*g*_, and *s*_0*g*_^2 ^is the estimated prior variance. As explained below, we chose to model the function *s*_0*g*_^2 ^(*α*_*g*_) using local regression. The use of local regression differs from the window method of Cyber-T in that the window method pools the standard deviation estimates of all genes in the window, whereas local regression uses a *weighted *average of the log-variances, where the weight for each gene *j *depends on the difference between the intensity of gene *j *and the intensity of the gene *g*, of interest. This relationship on its own can significantly reduce the uncertainty in the true variance of gene expression variances. For example, the relationship shown in Figure 1 explains approximately 34% of variability in individual gene expression variances.

For our intensity-based method, we follow a hierarchical Bayesian set-up similar to SMT [12]. Individual gene variances for genes with similar overall expression levels are assumed to have been generated by a single probability distribution. The parameters for the distribution of the variances, *d*_0 _and *s*_0*g*_^2^, are termed the hyperparameters, and are estimated from the data using EB theory. In terms of the precision of the gene expression levels, which is defined as the reciprocal of the variance, 1/*s*_0*g*_^2 ^is the mean, andthe hyperparameter *d*_0 _is the prior degrees of freedom and determines the spread of the distribution for a given *s*_0*g*_^2^. Larger *d*_0 _values result in smaller spread of the distribution for the precision and variance of gene expression levels. Similar to previous methods [3,11], by assuming a single hyperparameter for the prior degrees of freedom, we make the assumption that the spread of variance estimates about the background variance level is similar across the entire range of fluorescence levels.

Suppose that β^
 MathType@MTEF@5@5@+=feaafiart1ev1aaatCvAUfKttLearuWrP9MDH5MBPbIqV92AaeXatLxBI9gBaebbnrfifHhDYfgasaacH8akY=wiFfYdH8Gipec8Eeeu0xXdbba9frFj0=OqFfea0dXdd9vqai=hGuQ8kuc9pgc9s8qqaq=dirpe0xb9q8qiLsFr0=vr0=vr0dc8meaabaqaciaacaGaaeqabaqabeGadaaakeaaiiGacuWFYoGygaqcaaaa@2E64@_*g *_is the estimate of the contrast of interest obtained after fitting the appropriate linear model for gene expression data for gene *g*. In the simplest case when comparing expression levels between two samples, β^
 MathType@MTEF@5@5@+=feaafiart1ev1aaatCvAUfKttLearuWrP9MDH5MBPbIqV92AaeXatLxBI9gBaebbnrfifHhDYfgasaacH8akY=wiFfYdH8Gipec8Eeeu0xXdbba9frFj0=OqFfea0dXdd9vqai=hGuQ8kuc9pgc9s8qqaq=dirpe0xb9q8qiLsFr0=vr0=vr0dc8meaabaqaciaacaGaaeqabaqabeGadaaakeaaiiGacuWFYoGygaqcaaaa@2E64@_*g *_is just the difference in average log-expression levels for gene *g *under the two experimental conditions. We assume the β^
 MathType@MTEF@5@5@+=feaafiart1ev1aaatCvAUfKttLearuWrP9MDH5MBPbIqV92AaeXatLxBI9gBaebbnrfifHhDYfgasaacH8akY=wiFfYdH8Gipec8Eeeu0xXdbba9frFj0=OqFfea0dXdd9vqai=hGuQ8kuc9pgc9s8qqaq=dirpe0xb9q8qiLsFr0=vr0=vr0dc8meaabaqaciaacaGaaeqabaqabeGadaaakeaaiiGacuWFYoGygaqcaaaa@2E64@_*g *_measurements of log-fold change for each gene follow a normal distribution centered at *β*_*g*_, the actual log-fold change:

β^
 MathType@MTEF@5@5@+=feaafiart1ev1aaatCvAUfKttLearuWrP9MDH5MBPbIqV92AaeXatLxBI9gBaebbnrfifHhDYfgasaacH8akY=wiFfYdH8Gipec8Eeeu0xXdbba9frFj0=OqFfea0dXdd9vqai=hGuQ8kuc9pgc9s8qqaq=dirpe0xb9q8qiLsFr0=vr0=vr0dc8meaabaqaciaacaGaaeqabaqabeGadaaakeaaiiGacuWFYoGygaqcaaaa@2E64@_*g *_~ *N *(*β*_*g*_, *v*_*g*_*σ*_*g*_^2^)

where *σ*_*g*_^2 ^is the residual variance in the linear model for gene *g *and *v*_*g *_is the coefficient of the variance required to calculate the standard error. For a two-sample *t*-test, *v*_*g *_is 1/*n*_1 _+ 1/*n*_2 _where *n*_1 _and *n*_2 _are the number of observations for each sample. Given the variance *σ*_*g*_^2^, the sample variance for each gene is assumed to follow a scaled Chi-square distribution with *d*_*g *_degrees of freedom:

sg2|σg2~σg2dgχdg2.
 MathType@MTEF@5@5@+=feaafiart1ev1aaatCvAUfKttLearuWrP9MDH5MBPbIqV92AaeXatLxBI9gBaebbnrfifHhDYfgasaacH8akY=wiFfYdH8Gipec8Eeeu0xXdbba9frFj0=OqFfea0dXdd9vqai=hGuQ8kuc9pgc9s8qqaq=dirpe0xb9q8qiLsFr0=vr0=vr0dc8meaabaqaciaacaGaaeqabaqabeGadaaakeaacqWGZbWCdaqhaaWcbaGaem4zaCgabaGaeGOmaidaaOGaeiiFaWhcciGae83Wdm3aa0baaSqaaiabdEgaNbqaaiabikdaYaaakiabc6ha+naalaaabaGae83Wdm3aa0baaSqaaiabdEgaNbqaaiabikdaYaaaaOqaaiabdsgaKnaaBaaaleaacqWGNbWzaeqaaaaakiab=D8aJnaaDaaaleaacqWGKbazdaWgaaadbaGaem4zaCgabeaaaSqaaiabikdaYaaakiabc6caUaaa@45B4@

We adopt the conjugate prior distribution for *σ*_*g*_^2^

1σg2~1d0s0g2χd02
 MathType@MTEF@5@5@+=feaafiart1ev1aaatCvAUfKttLearuWrP9MDH5MBPbIqV92AaeXatLxBI9gBaebbnrfifHhDYfgasaacH8akY=wiFfYdH8Gipec8Eeeu0xXdbba9frFj0=OqFfea0dXdd9vqai=hGuQ8kuc9pgc9s8qqaq=dirpe0xb9q8qiLsFr0=vr0=vr0dc8meaabaqaciaacaGaaeqabaqabeGadaaakeaadaWcaaqaaiabigdaXaqaaGGaciab=n8aZnaaDaaaleaacqWGNbWzaeaacqaIYaGmaaaaaOGaeiOFa43aaSaaaeaacqaIXaqmaeaacqWGKbazdaWgaaWcbaGaeGimaadabeaakiabdohaZnaaDaaaleaacqaIWaamcqWGNbWzaeaacqaIYaGmaaaaaOGae83Xdm2aa0baaSqaaiabdsgaKnaaBaaameaacqaIWaamaeqaaaWcbaGaeGOmaidaaaaa@4114@

where *d*_0 _and *s*_0*g*_^2 ^are the hyperparameters for the degrees of freedom and variance, respectively. With this model, the closed-form solutions for the posterior mean of the variance and degrees of freedom given the hyperparameters are:

df=d0+dgs˜g2=d0s0g2+dgsg2d0+dg
 MathType@MTEF@5@5@+=feaafiart1ev1aaatCvAUfKttLearuWrP9MDH5MBPbIqV92AaeXatLxBI9gBaebbnrfifHhDYfgasaacH8akY=wiFfYdH8Gipec8Eeeu0xXdbba9frFj0=OqFfea0dXdd9vqai=hGuQ8kuc9pgc9s8qqaq=dirpe0xb9q8qiLsFr0=vr0=vr0dc8meaabaqaciaacaGaaeqabaqabeGadaaakqaabeqaaiabdsgaKjabdAgaMjabg2da9iabdsgaKnaaBaaaleaacqaIWaamaeqaaOGaey4kaSIaemizaq2aaSbaaSqaaiabdEgaNbqabaaakeaacuWGZbWCgaacamaaDaaaleaacqWGNbWzaeaacqaIYaGmaaGccqGH9aqpdaWcaaqaaiabdsgaKnaaBaaaleaacqaIWaamaeqaaOGaem4Cam3aa0baaSqaaiabicdaWiabdEgaNbqaaiabikdaYaaakiabgUcaRiabdsgaKnaaBaaaleaacqWGNbWzaeqaaOGaem4Cam3aa0baaSqaaiabdEgaNbqaaiabikdaYaaaaOqaaiabdsgaKnaaBaaaleaacqaIWaamaeqaaOGaey4kaSIaemizaq2aaSbaaSqaaiabdEgaNbqabaaaaaaaaa@50D4@

where *df *is the posterior degrees of freedom, *d*_*g *_is likelihood degrees of freedom, and s˜g2
 MathType@MTEF@5@5@+=feaafiart1ev1aaatCvAUfKttLearuWrP9MDH5MBPbIqV92AaeXatLxBI9gBaebbnrfifHhDYfgasaacH8akY=wiFfYdH8Gipec8Eeeu0xXdbba9frFj0=OqFfea0dXdd9vqai=hGuQ8kuc9pgc9s8qqaq=dirpe0xb9q8qiLsFr0=vr0=vr0dc8meaabaqaciaacaGaaeqabaqabeGadaaakeaacuWGZbWCgaacamaaDaaaleaacqWGNbWzaeaacqaIYaGmaaaaaa@30A0@ is the posterior mean of the variance. Our goal is to calculate point estimates of hyperparameters so that we can calculate expected values for the posterior parameters, *σ*_*g*_^2 ^and *df*.

We can now use the moderated t-statistic:

tgi=β^gis˜gvgi
 MathType@MTEF@5@5@+=feaafiart1ev1aaatCvAUfKttLearuWrP9MDH5MBPbIqV92AaeXatLxBI9gBaebbnrfifHhDYfgasaacH8akY=wiFfYdH8Gipec8Eeeu0xXdbba9frFj0=OqFfea0dXdd9vqai=hGuQ8kuc9pgc9s8qqaq=dirpe0xb9q8qiLsFr0=vr0=vr0dc8meaabaqaciaacaGaaeqabaqabeGadaaakeaacqWG0baDdaWgaaWcbaGaem4zaCMaemyAaKgabeaakiabg2da9maalaaabaacciGaf8NSdiMbaKaadaWgaaWcbaGaem4zaCMaemyAaKgabeaaaOqaaiqbdohaZzaaiaWaaSbaaSqaaiabdEgaNbqabaGcdaGcaaqaaiabdAha2naaBaaaleaacqWGNbWzcqWGPbqAaeqaaaqabaaaaaaa@3E29@

to test the hypothesis H_0_: *β*_*g *_= 0 vs. H_A_: *β*_*g *_≠ 0 with *df *degrees of freedom, where β^
 MathType@MTEF@5@5@+=feaafiart1ev1aaatCvAUfKttLearuWrP9MDH5MBPbIqV92AaeXatLxBI9gBaebbnrfifHhDYfgasaacH8akY=wiFfYdH8Gipec8Eeeu0xXdbba9frFj0=OqFfea0dXdd9vqai=hGuQ8kuc9pgc9s8qqaq=dirpe0xb9q8qiLsFr0=vr0=vr0dc8meaabaqaciaacaGaaeqabaqabeGadaaakeaaiiGacuWFYoGygaqcaaaa@2E64@_*gi *_is the estimate of log-fold change for gene *g *and contrast *i*, and s˜
 MathType@MTEF@5@5@+=feaafiart1ev1aaatCvAUfKttLearuWrP9MDH5MBPbIqV92AaeXatLxBI9gBaebbnrfifHhDYfgasaacH8akY=wiFfYdH8Gipec8Eeeu0xXdbba9frFj0=OqFfea0dXdd9vqai=hGuQ8kuc9pgc9s8qqaq=dirpe0xb9q8qiLsFr0=vr0=vr0dc8meaabaqaciaacaGaaeqabaqabeGadaaakeaacuWGZbWCgaacaaaa@2E2A@_*g *_is the posterior standard deviation.

As demonstrated by Smyth [12], under the null-hypothesis, the resulting moderated T-statistic in IBMT is distributed as Student's-t with *df *degrees of freedom. Thus, differentially expressed genes can be identified by calculating *p*-values and making appropriate multiple comparisons adjustments. However, if the data grossly deviate from the distributional assumptions, the moderated t-statistics can be used as a heuristic score for ranking genes based on the likelihood that they are differentially expressed, or an alternative empirical-based multiple comparison adjustment can be made, as in [21].

### Estimation of hyperparameters

The formulas for posterior mean of the variance and degrees of freedom assume known hyperparameters *d*_0 _and *s*_0*g*_. We follow the empirical Bayes approach and estimate hyperparameters from the data. Gene-specific prior variances are estimated from *f*(*α*_*g*_) as given in (1), where *f*(·) is a fitted local regression model of adjusted individual genes' log-variances (see equation 4) on the average log-expression levels. In this way, we avoid having to pre-specify a functional form for this dependency, and obtain predicted variances for each gene given their spot intensities.

To estimate the prior variance and prior degrees of freedom, we use the common empirical Bayesian method of equating the empirical to expected values for the first and second moments of log-variance. According to the hierarchical model, the sampling variance for each gene, marginally, has the following scaled-F distribution [12]:

sg2
 MathType@MTEF@5@5@+=feaafiart1ev1aaatCvAUfKttLearuWrP9MDH5MBPbIqV92AaeXatLxBI9gBaebbnrfifHhDYfgasaacH8akY=wiFfYdH8Gipec8Eeeu0xXdbba9frFj0=OqFfea0dXdd9vqai=hGuQ8kuc9pgc9s8qqaq=dirpe0xb9q8qiLsFr0=vr0=vr0dc8meaabaqaciaacaGaaeqabaqabeGadaaakeaacqWGZbWCdaqhaaWcbaGaem4zaCgabaGaeGOmaidaaaaa@3091@ ~ s0g2
 MathType@MTEF@5@5@+=feaafiart1ev1aaatCvAUfKttLearuWrP9MDH5MBPbIqV92AaeXatLxBI9gBaebbnrfifHhDYfgasaacH8akY=wiFfYdH8Gipec8Eeeu0xXdbba9frFj0=OqFfea0dXdd9vqai=hGuQ8kuc9pgc9s8qqaq=dirpe0xb9q8qiLsFr0=vr0=vr0dc8meaabaqaciaacaGaaeqabaqabeGadaaakeaacqWGZbWCdaqhaaWcbaGaeGimaaJaem4zaCgabaGaeGOmaidaaaaa@317F@*F*_*dg*,*d*0_

Consequently, the log-sample variance is distributed as the sum of a constant and Fisher's Z distribution and has the following expected value and variance:

*E *(log sg2
 MathType@MTEF@5@5@+=feaafiart1ev1aaatCvAUfKttLearuWrP9MDH5MBPbIqV92AaeXatLxBI9gBaebbnrfifHhDYfgasaacH8akY=wiFfYdH8Gipec8Eeeu0xXdbba9frFj0=OqFfea0dXdd9vqai=hGuQ8kuc9pgc9s8qqaq=dirpe0xb9q8qiLsFr0=vr0=vr0dc8meaabaqaciaacaGaaeqabaqabeGadaaakeaacqWGZbWCdaqhaaWcbaGaem4zaCgabaGaeGOmaidaaaaa@3091@) = log s0g2
 MathType@MTEF@5@5@+=feaafiart1ev1aaatCvAUfKttLearuWrP9MDH5MBPbIqV92AaeXatLxBI9gBaebbnrfifHhDYfgasaacH8akY=wiFfYdH8Gipec8Eeeu0xXdbba9frFj0=OqFfea0dXdd9vqai=hGuQ8kuc9pgc9s8qqaq=dirpe0xb9q8qiLsFr0=vr0=vr0dc8meaabaqaciaacaGaaeqabaqabeGadaaakeaacqWGZbWCdaqhaaWcbaGaeGimaaJaem4zaCgabaGaeGOmaidaaaaa@317F@ + *ψ*(*d*_*g*_/2) - *ψ*(*d*_0_/2) + log(*d*_0_/*d*_*g*_)     (2)

var(log sg2
 MathType@MTEF@5@5@+=feaafiart1ev1aaatCvAUfKttLearuWrP9MDH5MBPbIqV92AaeXatLxBI9gBaebbnrfifHhDYfgasaacH8akY=wiFfYdH8Gipec8Eeeu0xXdbba9frFj0=OqFfea0dXdd9vqai=hGuQ8kuc9pgc9s8qqaq=dirpe0xb9q8qiLsFr0=vr0=vr0dc8meaabaqaciaacaGaaeqabaqabeGadaaakeaacqWGZbWCdaqhaaWcbaGaem4zaCgabaGaeGOmaidaaaaa@3091@) = *ψ*'(*d*_*g*_/2) + *ψ*'(*d*_0_/2)     (3)

where *ψ*() is the digamma function and *ψ*'() is the trigamma function [12,22]. We denote with *e*_*g *_the non-constant part of (2) for each gene after solving for log(*s*_0*g*_^2^)

*e*_*g *_= log sg2
 MathType@MTEF@5@5@+=feaafiart1ev1aaatCvAUfKttLearuWrP9MDH5MBPbIqV92AaeXatLxBI9gBaebbnrfifHhDYfgasaacH8akY=wiFfYdH8Gipec8Eeeu0xXdbba9frFj0=OqFfea0dXdd9vqai=hGuQ8kuc9pgc9s8qqaq=dirpe0xb9q8qiLsFr0=vr0=vr0dc8meaabaqaciaacaGaaeqabaqabeGadaaakeaacqWGZbWCdaqhaaWcbaGaem4zaCgabaGaeGOmaidaaaaa@3091@ - *ψ*(*d*_*g*_/2) + log(*d*_*g*_/2),     (4)

with

*E*(*e*_*g*_) = log s0g2
 MathType@MTEF@5@5@+=feaafiart1ev1aaatCvAUfKttLearuWrP9MDH5MBPbIqV92AaeXatLxBI9gBaebbnrfifHhDYfgasaacH8akY=wiFfYdH8Gipec8Eeeu0xXdbba9frFj0=OqFfea0dXdd9vqai=hGuQ8kuc9pgc9s8qqaq=dirpe0xb9q8qiLsFr0=vr0=vr0dc8meaabaqaciaacaGaaeqabaqabeGadaaakeaacqWGZbWCdaqhaaWcbaGaeGimaaJaem4zaCgabaGaeGOmaidaaaaa@317F@ - *ψ*(*d*_0_/2) + log(*d*_0_/2).     (5)

Next, we determine the predicted values for *e*_*g*_, *pred*(*e*_*g*_), as a function of average log-intensities by local regression. We define the prior variance for each gene, *s*_0*g*_^2^, to be the exponential of *pred*(*e*_*g*_) + *ψ*(*d*_0_/2) - log(*d*_0_/2), by substituting *pred*(*e*_*g*_) for *E*(*e*_*g*_) in (5) and solving for log(*s*_0*g*_^2^). To calculate the prior degrees of freedom we equate the empirical variance of the log-sample variances with the marginal variance in (3) and solve for *d*_0_. As indicated before, we assume *a priori *that *σ*_*g*_^2 ^varies with *g*, but its variance is constant for all *g*. Thus, if *d*_*g*_'s were all the same and *ψ*'(*d*_*g*_/2) = *c*, say, then the marginal variance as given in (3) would be a constant, with a consistent estimator given by

mean[eg−pred(eg)]2=1n∑[eg−pred(eg)]2.
 MathType@MTEF@5@5@+=feaafiart1ev1aaatCvAUfKttLearuWrP9MDH5MBPbIqV92AaeXatLxBI9gBaebbnrfifHhDYfgasaacH8akY=wiFfYdH8Gipec8Eeeu0xXdbba9frFj0=OqFfea0dXdd9vqai=hGuQ8kuc9pgc9s8qqaq=dirpe0xb9q8qiLsFr0=vr0=vr0dc8meaabaqaciaacaGaaeqabaqabeGadaaakeaacqWGTbqBcqWGLbqzcqWGHbqycqWGUbGBcqGGBbWwcqWGLbqzdaWgaaWcbaGaem4zaCgabeaakiabgkHiTiabdchaWjabdkhaYjabdwgaLjabdsgaKjabcIcaOiabdwgaLnaaBaaaleaacqWGNbWzaeqaaOGaeiykaKIaeiyxa01aaWbaaSqabeaacqaIYaGmaaGccqGH9aqpdaWcaaqaaiabigdaXaqaaiabd6gaUbaadaaeabqaaiabcUfaBjabdwgaLnaaBaaaleaacqWGNbWzaeqaaaqabeqaniabggHiLdGccqGHsislcqWGWbaCcqWGYbGCcqWGLbqzcqWGKbazcqGGOaakcqWGLbqzdaWgaaWcbaGaem4zaCgabeaakiabcMcaPiabc2faDnaaCaaaleqabaGaeGOmaidaaOGaeiOla4caaa@5B6E@

This would yield an estimator for *ψ*'(*d*_0_/2), given by

*mean*[*e*_*g *_- *pred*(*e*_*g*_)]^2 ^- *c*.     (6)

When *d*_*g*_'s are different, the marginal variances in (3) differ for different *g*, but by known values *ψ*'(*d*_*g*_/2). Thus if we assume that *d*_*g *_does not vary drastically, in the sense that *mean*[*ψ*'(*d*_*g*_/2)] = (1/*n*)∑*ψ*'(*d*_*g*_/2) approaches a constant *c *as *n *gets large, then (6) is a consistent estimate of *ψ*'(*d*_0_/2). Typically, *d*_*g *_does not vary substantially with good quality data, and with Affymetrix data *d*_*g *_is usually constant. Thus *d*_0 _can be estimated consistently by solving

*ψ*'(*d*_0_/2) = *mean*[*e*_*g *_- *pred*(*e*_*g*_)]^2 ^- *mean*[*ψ*'(*d*_*g*_/2)]

for *d*_0_. Note that if *d*_*g *_is constant for all genes, then using *log s*_*g*_^2 ^in placement of *e*_*g *_results in the same solution for *d*_0_.

### Simulation study

**Figure 2:**
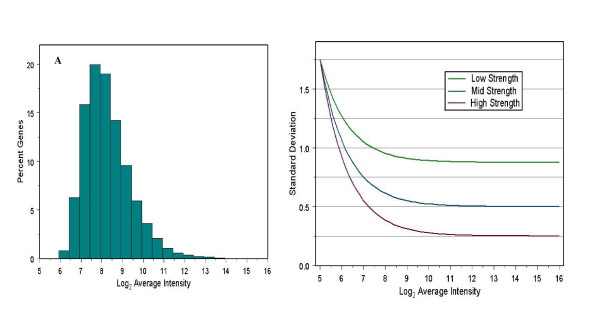
**Values used in simulations**. (A) Distribution of average log-expression levels. (B) Three strengths of dependency of gene standard deviation on expression intensity used in simulations.

Simulations were designed to imitate a six slide, single-channel microarray experiment with three treatments and three controls. The simulations were performed to compare the performance of five methods (*t*-test, fold change, SMT, IBMT, and Fox) with respect to: a) the strength of relationship between variance and signal intensity, b) estimation of the correct prior degrees of freedom, and c) unbiased estimation of the true false positive rate. Average expression intensities were generated assuming a log-normal distribution with a scale parameter of 1.1, shape parameter equal to 0.34, and threshold parameter 5.1. The parameters for this distribution were chosen to closely fit the actual distribution of average expression intensities seen from real experiments (Figure 2a). Simulations were run assuming prior degrees of freedom *d*_0 _∈ [1, 4, 16, 100]. For each prior degrees of freedom, actual and sample standard deviations were simulated for three different strengths of dependency on average log-intensities (Figure 2b), referred to as low, medium, and high. The specific functional form used for this was

*g*(*x*) = *p*_1_*e*^-0.8(*x*-5) ^+ *p*_2_

with the following values used for *p*_1 _and *p*_2_: low: *p*_1 _= *p*_2 _= 0.875, medium: *p*_1 _= 1.25 and *p*_2 _= 0.5, and high: *p*_1 _= 1.5, *p*_2 _= 0.25. To determine differences among the methods due to sample size, additional simulations were run for a 4-slide experiment (two treatment, two control) and a 10-slide experiment (five treatment, five control), with the high strength dependency, and an additional simulation was also run for the 6-slide experiment with no dependency of variance on average intensities. In the case of no dependence, IBMT performed nearly identical to SMT. All simulations were performed with 15000 "genes", 300 (2%) of which were designed to be "differentially expressed". Log-ratios for all genes were simulated as described in [12]. Actual mean log-ratios for the 300 differentially expressed genes were simulated from the normal distribution N(0, 3•*σ*_*g*_^2^), and simulated *measured *mean log-ratios for all genes were assumed to follow the normal distribution N(*μ*, *σ*_*g*_^2^/3), where *μ *= 0 if the gene is not differentially expressed, and the simulated log-ratio for the 300 (2%) differentially expressed genes.

The simulation process is summarized here:

For all 15000 genes:

1. Simulate *α*_*g *_as random draws from a log-normal distribution,

2. Define function, *f*(*α*_*g*_), for dependence of variance on *α*_*g*,_

3. Simulate *σ*_*g*_^2 ^as random draws from *d*_0_*f(*α*_*g*_)/(chi-square with *d*_0 _degrees of freedom),

4. Simulate *s*_*g*_^2 ^as random draws from *σ*_*g*_^2^/*d*_g_*chi-square with *d*_*g *_= 4 degrees of freedom,

5. W.L.O.G., assume the first 300 genes are differentially expressed,

Simulate their mean log-ratios *μ*_*g *_as random draws from N(0, 3*σ*_*g*_^2^),

6. For the remaining 14700 non-differentially expressed genes

Set *μ*_*g *_= 0,

7. Simulate estimated log-ratios as random draws from N(*μ*_*g*_, *σ*_*g*_^2^/3).

**Figure 3:**
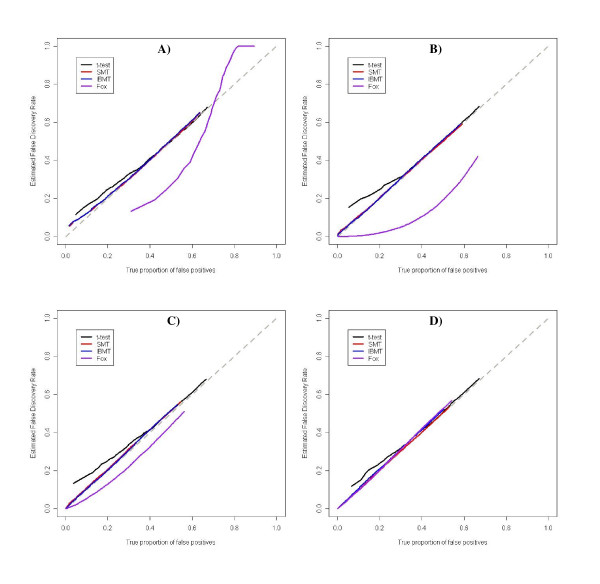
**IBMT correctly estimates the proportion of false positives**. All tested methods except Fox (*t*-test, SMT, and IBMT) correctly control for the true false positive rate. Data shown is the average of 100 simulations and the mid-strength dependence of variance on expression level with **(A) ***d*_*g *_= 4, *d*_0 _= 1, **(B) ***d*_*g *_= 4, *d*_0 _= 4, **(C) ***d*_*g *_= 4, *d*_0 _= 16, and **(D) ***d*_*g *_= 4, *d*_0 _= 100.

Results from the simulations indicate that the added complexity of the model is outweighed by the additional gain in information. Four methods were compared in their ability to correctly estimate the false positive rate, using estimated False Discovery Rates (FDR) [23]: the simple T-statistic (T), Smyth's moderated T-statistic (SMT), our intensity-based moderated-T (IBMT) method, and Fox's method (Fox). All methods except Fox accurately estimate the percent of false positives, as demonstrated by Figure 3. When the prior degrees of freedom is low, Fox's method underestimates the percent of false positives (Figure 3a and 3b), suggesting the possibility of a real risk of Fox's method to give overly-optimistic results with real data. Control of the true false positive rate under additional parameter sets gave the same results, and may be viewed as Supplemental Figure S2 [see Additional file 1].

**Figure 4:**
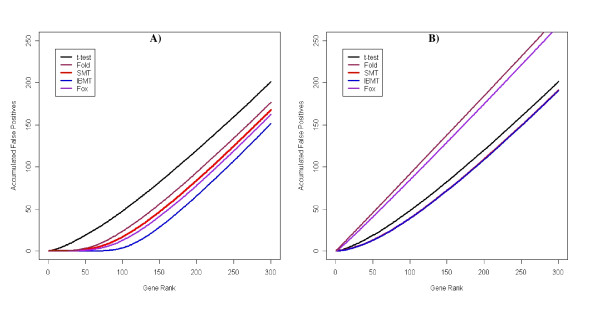
**Example false positive curves**. Number of falsely implicated differentially expressed genes with rank ≤ *x *for the simple *t*-test, fold change cut-off, SMT, Fox, and IBMT methods. Figure shows the accumulation of false positives by gene rank. Data shown is the average of 100 simulations using **(A) **the high-strength dependence of variance on expression level and 100 prior degrees of freedom, and **(B) **the mid-strength dependence and 1 prior degree of freedom.

**Figure 5:**
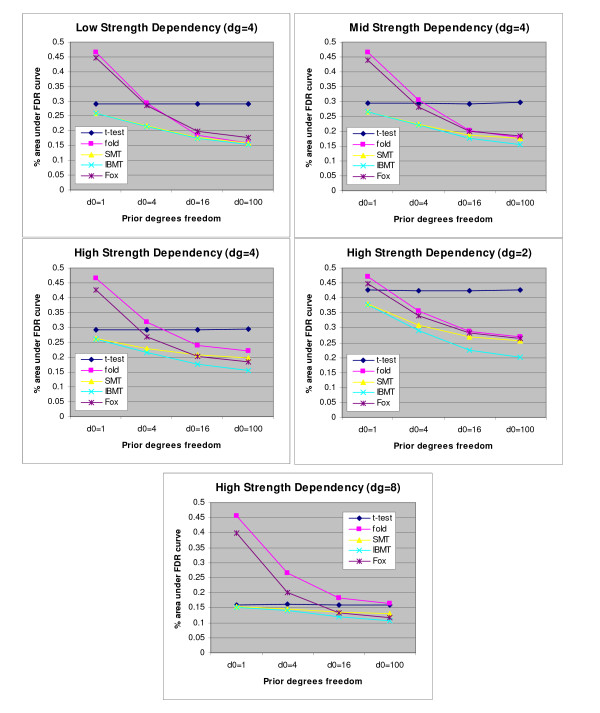
**Areas under false positive curves for all three strengths of dependency of variance on average spot intensity, and for additional simulations**. Areas are normalized so that the highest (worst) possible area is 0.50, the lowest (best) being 0.00. **(A) **Low strength dependency- the fold change method performed poorest for low prior degrees of freedom, while the simple *t*-test is poorest with high prior degrees of freedom. IBMT performs minimally better than SMT in this case. Fox performs similarly to fold change **(B) **Medium strength dependency- Similar to above, but with the advantage of IBMT larger for high prior degrees of freedom **(C) **High strength dependency- IBMT performs better than all other methods, especially for mid to high prior degrees of freedom. **(D) **4-slide simulation- Similar to (C), but with overall poorer performance by the *t*-test, and slightly more advantage by IBMT. **(E) **10-slide simulation- Fox now performs significantly better than fold change, but both have very poor performance for low prior degrees of freedom. IBMT still performs best.

We compared the ability of the methods to identify differentially expressed genes by creating false positive rate curves for each parameter set. These were created by ranking the genes by significance level, and then calculating the number of accumulated false positives with rank less than or equal to *x*. Example false positive rate curves for the five methods are shown in Figure 4. Figure 5 summarizes the results for all parameter sets by presenting normalized areas under the false positive curves described above. All results shown are the average of 100 simulation runs. All methods performed poorly when the data was simulated with only one prior degree of freedom. As the number of prior degrees of freedom increased, the performance of all methods except the simple *t*-test improved with IBMT overall outperforming the other methods. Fox's method closely followed the performance of the fold change method, with a substantial advantage over fold with high dependence of variance on signal intensity. However, it had poor performance when gene's variances were approximately independent (small prior degrees of freedom). Both these results are probably due to this method's assumption that genes with similar intensities have identical variances. For the simulation with no dependence of variance on expression level, the areas under the false positive curves were the same for both SMT and IBMT. The poor performance of the simple T-statistic in these simulations is most likely related to the low number of experimental replicates. We used four sample degrees of freedom, which was insufficient to accurately measure the variance of each gene separately. In additional simulations performed with higher sample degrees of freedom (8, 12, and 16), the simple *t*-test showed marked improvement over results based on fewer degrees of freedom, while the other methods did not show as much improvement as the degrees of freedom increased (supplemental Figure S3).

Finally we compared the ability of IBMT to SMT to accurately estimate the prior degrees of freedom (Table 1). Since Fox's prior degrees of freedom is dependent only on the free parameter and sample size rather than estimated from the data (default *d*_0 _= 16 for all 4-slide simulations), Fox was not included in this comparison. As expected, the empirical Bayes method that does not account for the relationship between the variance and the magnitude of expression measurements tends to underestimate the prior degrees of freedom, especially for larger *d*_0 _values. As the dependency of variance on average intensities increases, this bias grows stronger. For the simulation with no dependence of variance on intensity level, using *d*_0 _= 16, both methods accurately estimated the prior degrees of freedom, with estimates of *d*_0_/(*d*_0_+*d*_*g*_) equal to 0.802 and 0.803 for SMT and IBMT respectively.

**Table 1:** Simulated estimation of prior degrees of freedom for SMT and IBMT

**Dependency strength of variance on intensity**	**Method**	**d_0 _= 1**	**d_0 _= 4**	**d_0 _= 16**	**d_0 _= 100**
**Low**	**SMT**	0.200	0.494	0.774	0.923
	**IBMT**	0.200	0.500	0.800	0.963
**Middle**	**SMT**	0.198	0.472	0.703	0.813
	**IBMT**	0.200	0.501	0.800	0.961
**High**	**SMT**	0.194	0.422	0.571	0.630
	**IBMT**	0.200	0.500	0.801	0.962

Values listed are for the function *d*_0_/(*d*_0 _+ *d*_*g*_) and are the mean of 100 simulations. Perfect values for each prior degrees of freedom are: **d_0 _= 1**: 0.20, **d_0 _= 4**: 0.50, **d_0 _= 16**: 0.80, and **d_0 _= 100**: 0.962

### Results from the controlled spike-in dataset

**Figure 6:**
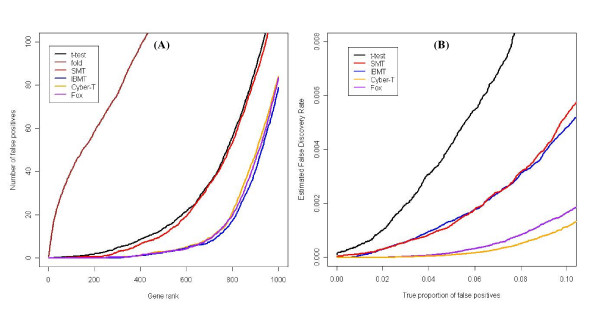
**Results from the Choe, et al. spike-inexperiment**. **(A) **IBMT results in the fewest false positives overall. The other methods, from best to worst, are Fox, Cyber-T, SMT, *t*-test, and fold change. **(B) **Comparison of how accurately each method estimates the true proportion of false positives. The simple *t*-test performs best in correctly estimating its false positive rate, although all methods underestimate the true number of false positives, as noted in [25]. Fox's method and especially Cyber-T result in the greatest underestimation of false positives.

Two publicly-available, and completely controlled, "spike-in" Affymetrix datasets were used to compare the performance of the same methods, plus Cyber-T, on real-world microarray data. The analysis of these experiments is a natural extension of the simulation studies as the "correct" results are known. The first experiment consisted of three technical replicates each of control RNA samples and samples with known amounts of spiked-in RNA, and consisting of 3,860 individual cRNAs. We used the average of the top 10 expression datasets, as reported by Choe et al. [14] and available for download at [24]. The description of all pre-processing steps used for these expression datasets, as well as further detail of the experimental methods are given in the original publication [3]. In the original publication, Cyber-T was determined to be the preferred method for identifying differentially expressed genes, with *SAM *[9] and the simple *t*-test being the other methods tested. For all six methods (*t*-test, fold, SMT, IBMT, Cyber-T, and Fox), we ranked the genes by significance level, and then the number of false positives was calculated as a function of the number of genes deemed to be significant. The order of performance in accumulating the least number of false positives, from best to worst, is IBMT, Fox, Cyber-T, SMT, the simple *t*-test, and finally fold change (Figure 6a).

The ability of the different methods to correctly establish the statistical significance of differential expression was assessed by comparing estimated and empirically established False Discovery Rates (FDR) [23]. The simple *t*-test performed best in correctly estimating the FDR (Figure 6b). Of the four other methods, IBMT and SMT resulted in estimated False Discovery rates closest to their true proportion of false positive rates (Figure 6b). All five methods underestimate the number of false positives, which under normal circumstances may result in an unacceptable amount of over-confidence in the significance of results. However, we stress that in this experiment even the simple *t*-test underestimated the true number of false positives, as has been previously noted [25]. The prior degrees of freedom estimated for this study ranged from 4.0 – 5.4 for IBMT and 1.6 – 1.9 for SMT, and using the defaults for the other methods, Cyber-T used 10 and Fox used 16.

**Figure 7:**
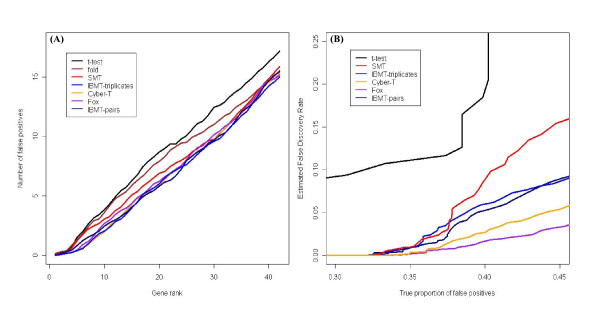
**Results from HG-U133 latin-square spike-in experiment**. **(A) **Methods that account for the dependency of variance on signal intensity (IBMT, Cyber-T, and Fox) accumulate the fewest false positives **(B) **The simple *t*-test performs best in estimating the true proportion of false positives, and the others from best to worst, are SMT, IBMT, Cyber-T, and Fox.

The second spike-in dataset used was the Affymetrix HG-U133 latin-square data set available at [26], and consisting of 22,300 probe sets. This dataset consists of 14 sets of 3 chips, each having 42 probe sets (0.19%) spiked-in. After preprocessing with RMA, each consecutive pair of triplicates was analyzed separately, to identify the 2-fold changes in expression. In addition, IBMT was used to analyze each set of three consecutive triplicates. Figure 7a and 7b compare the average accumulation of false positives by gene rank and estimation of the true proportion of false positives respectively. Note the slight improvement in using three sets at a time compared to pairs. Possibly due to the low number of spiked-in genes for this experiment, the ability of IBMT, Cyber-T, and Fox to rank the differentially expressed genes on top could not be differentiated, as the curves for these three experiments cross. However, these methods did outperform SMT, fold change, and the *t*-test, again indicating the importance of accounting for the dependence of variance on gene signal intensity. Similar to the previous spike-in experiment, Figure 7b shows that the *t*-test performed best in estimating the true proportion of false positives, and Cyber-T and Fox resulted in the greatest underestimation of false positives. Prior degrees of freedom for this data set ranged from 7.6 – 19.3 for IBMT and 5.2 – 8.2 for SMT, while Cyber-T and Fox used the same defaults as the previous data set. The relationship between variance and intensity for this study can be seen in Supplemental Figure S4.

### Case studies: analysis and interpretation of two microarray datasets

#### Results from the MEF Ahr^-/- ^dataset

Although simulations and spike-in datasets point to the potential advantage of IBMT and allow a determination of its general behavior, only with the analysis of experimental data can the practical advantages or disadvantages of the method be observed. We compared the *t*-test based on the simple linear model, fold change cut-off, SMT, and IBMT on two experimental datasets. Cyber-T and Fox's method were not included because they could not be properly used with the experimental designs of these datasets. The first is a comparison of relative RNA levels of *wildtype *mouse embryo fibroblast (MEF) cells to aryl-hydrocarbon receptor gene (*Ahr*) knockout MEF cells, involving both technical and biological replicate arrays. The aryl-hydrocarbon receptor protein (AHR) is a critical mediator of the molecular defense of exposures to environmental toxicants by serving as the receptor in a toxicant-activated signaling pathway [27]. The top 300 (2.2%) ranked genes from each of the four methods were used to test for Gene Ontology categories significantly enriched with differentially expressed genes to compare the ability of each method to reveal pathways or cellular processes involved in AHR function. We used a fixed number of genes to test Gene Ontology to keep the comparison of methods unbiased. Testing was performed using Expression Analysis Systematic Explorer (EASE), and linking to the three branches of the Gene Ontology database. Fisher's Exact probability was calculated for each gene category, and a Bonferroni-adjusted *p*-value < 0.1 was used as the significance cut-off level [28]. Assuming the treatment affects a certain number of known biological pathways and molecular functions in the cell, the method that detects the highest number of these is the most desirable.

Table 2 shows the top 10 significant Gene Ontology categories for each method. IBMT had the highest number (17) of significant categories as well as the highest number of unique genes (144) involved in those categories. All four methods identified extracellular genes and genes involved in the extracellular space as important categories altered when the *Ahr *gene is knocked-out. This is consistent with what has previously been observed in vascular SMCs [16]. IBMT further recognized "response to external stimulus" (as well as several of its progeny: response to biotic stimulus, defense response, and immune response) as being significantly affected. Once the AHR is activated by the binding of an exogenous toxicant, the AHR induces the transcriptional activity of a battery of xenobiotic metabolizing genes as part of a host defensive response [29] and interacts with other signaling pathways to either stimulate or depress signal transduction [30]. In addition, the interaction of the AHR and TGF-*β *signalling pathways is known to greatly affect those genes that encode extracelluar matix (ECM) and ECM remodeling proteins [16]. The full list of significant categories and top ranked genes from each method are available as supplemental information [see Additional file 1].

**Table 2:** Top significant Gene Ontology categories for the MEF Ahr^-/- ^dataset

***Top 10 GO***	***t*-test**	**FOLD**	**SMT**	**IBMT**
1	Extracellular space (77)	Extracellular (91)	Extracellular (90)	Extracellular (92)
2	Extracellular (84)	Extracellular space (82)	Extracellular space (81)	Response to biotic stimulus (39)
3	Integrin binding (5)	Signal transducer activity (67)	Receptor binding (27)	Extracellular space (80)
4	Spermine/Spermidine biosynthesis (3)	Organogenesis (38)	Chemoattractant activity (8)	Response to external stimulus (46)
5	Carboxy peptidase activity (6)	Chemoattractant activity (7)	Signal transducer activity (68)	Defense response (34)
6	Spermidine metabolism (3)	Receptor binding (24)	Response to biotic stimulus (33)	Signal transducer activity (68)
7	Polyamine biosynthesis (3)	Histogenesis and organogenesis (9)	Chemokine receptor binding/activity (7)	Chemoattractant activity (8)
8	Receptor binding (22)	Morphogenesis (39)	Integrin binding (5)	Immune response (27)
9	Adenosylmethionine decarboxylase activity (2)	Serine-type endopeptidase inhibitor activity (9)	G-protein-coupled receptor binding (7)	Response to pest/pathogen/parasite (19)
10	Spermine metabolism (3)	glycosaminoglycan binding (7)	Spermine/Spermidine biosynthesis (3)	Chemokine receptor binding/activity (7)
# Bonf<0.1	6	8	13	17
# genes ↑	92	142	135	144

Top ten categories for each of the four compared methods: magnitude of fold change, simple *t*-test, SMT, and IBMT. The IBMT method resulted in both the highest number of significant categories using a 0.10 Bonferroni-adjusted *p*-value cut-off, as well as the highest number of genes in a significant category.

#### Results from Nickel exposure dataset

The second experimental dataset that we analysed using IBMT is a time series response to nickel inhalation in female 129S1/SvImJ strain mouse lung [19]. Five times were used (3, 8, 24, 48, and 72 hours), each being compared to control samples in triplicate. For each time, samples for one array were labelled with opposite dyes. Data was normalized and analysed for differentially expressed genes as described in the methods. As in the previous section, the analysis of this experiment, which must account for both dye-effect and multiple treatment conditions, is an example not able to be analysed correctly by either Cyber-T or Fox's method.

We tested for significant GO categories as described above for the top ranked 200 (1.5%) genes in each comparison, and three different *p*-value cut-off values were used for significance rather than the stricter Bonferroni adjustment due to overall lower *p*-values from Fisher's Exact Test in this dataset. Two hundred rather than 300 genes were used in this experiment because only approximately 200 genes were significantly differentially expressed at the earliest time-point based on previous analysis. Table 3 displays a summary of the results from testing for significant Gene Ontology categories. IBMT found the highest number of unique genes (666) involved in the significantly found categories across time. The FOLD method results in the highest number of significant categories overall, and IBMT found the most significant categories using the two smaller *p*-values of 0.0001 and 0.001.

**Table 3:** Number of significant Gene Ontology categories and assigned genes among methods for Nickel exposure dataset

		**Number of unique genes**				**Number of significant categories**			
***p*-value**	**Time pt**	**T**	**FOLD**	**SMT**	**IBMT**	**T**	**FOLD**	**SMT**	**IBMT**

0.0001	03 hr	0	0	0	0	0	0	0	0
0.001	03 hr	0	0	0	0	0	0	0	0
0.005	03 hr	4	6	6	**8**	2	3	3	**4**
0.0001	08 hr	0	16	0	**46**	0	1	0	**2**
0.001	08 hr	0	49	12	**54**	0	5	9	**12**
0.005	08 hr	14	**71**	53	54	6	**28**	21	20
0.0001	24 hr	25	22	**26**	**26**	11	**15**	**15**	**15**
0.001	24 hr	52	32	**62**	56	15	19	**21**	19
0.005	24 hr	65	66	**72**	69	25	34	**35**	**35**
0.0001	48 hr	0	0	42	**46**	0	0	1	**2**
0.001	48 hr	2	9	44	**52**	1	3	4	**6**
0.005	48 hr	49	34	49	**60**	8	**26**	15	25
0.0001	72 hr	0	**59**	57	58	0	**9**	5	7
0.001	72 hr	45	61	63	**66**	3	**17**	12	11
0.005	72 hr	51	68	**77**	71	7	**42**	20	17

	Total	307	493	563	**666**	78	**202**	161	175
	# Zeroes	6	3	3	**2**				
	# Best	0	2	4	**8**	0	6	3	**7**

The number of significant categories, as well as the number of genes assigned to the significant categories, are shown for the five time points for each of three *p*-value cut-offs.

Given the nature of this experiment, one would expect that some functional categories would be affected at two or more time points. Therefore, an additional measure of performance is the level of overlap across time points in which categories were found to be significant. To accomplish this aim, we calculated the average number of time points each significant category was determined to be significant using the three same *p*-value cut-offs as above. The results are, for *p*-values of 0.0001, 0.001, and 0.005 respectively, FOLD: 1.04, 1.16, and 1.39; T: 1.00, 1.12, and 1.26; SMT: 1.17, 1.44, and 1.45; and IBMT: 1.30, 1.60, and 1.58. Thus, according to the results, the IBMT method gave the most consistent results through time. The list of significant GO categories is available as supplemental information [see Additional file 1].

Acute lung injury is a severe clinical syndrome that results from multiple causes including pneumonia, sepsis, trauma, and inhaled irritants [31]. Pathological conditions associated with the development of acute lung injury include alveolar damage, inflammatory cell influx and activation, pulmonary edema and hemorrhage, alteration of surfactant production, and insufficient gas exchange [31-33]. Prior studies have assessed aspects of the molecular mechanisms involved in the pathogenesis of acute lung injury in mice using inhaled nickel [19,34-40].

IBMT identified several transcripts that could play significant roles in the development of nickel-induced acute lung injury that were not recognized using the SMT method. For example, following 24 h of nickel exposure, transcripts for three heat shock proteins (HSPs) were found to be induced using the IBMT method as compared to the SMT method, including heat shock 70 kD protein 5 (HSPA5, 2.3-fold), heat shock protein 1B (HSPA1B, 2.4-fold), and heat shock protein 9A (HSPA9A, 2.3-fold). HSPs are a group of genes that are transcriptionally regulated in response to cellular stress. In the lung, induction of HSPs protects against acute lung injury in *in vivo *[41,42] and *in vitro *models [43-45]. Thus, HSP induction in response to nickel may be involved in an early cytoprotective mechanism in the development of acute lung injury.

Another transcript that was determined to be significantly changed using the IBMT method as compared to the SMT method was from a group of genes known as aquaporins, which facilitate water movement through the air space-capillary barrier in the lung [46]. Expression of aquaporin 5 (*Aqp5*), the major water channel gene expressed in alveolar, and bronchial epithelium, decreased an estimated 2.3-fold after 48 h of nickel exposure. In previous studies, decreased expression of *Aqp5 *has been associated with acute lung injury caused by adenoviral infection [47] and bleomycin treatment [48] in mice. These data are consistent with the modulation of *Aqp5 *expression in regulating fluid homeostasis and abnormal fluid fluxes in the development of pulmonary inflammation and edema associated with acute lung injury.

Finally, another significantly altered transcript that was identified by IBMT and not SMT was fibroblast growth factor 2 (FGF2, a.k.a. basic fibroblast growth factor). Mouse lung FGF2 transcript levels were estimated to be induced 5.6-fold after 72 h of nickel exposure. In the lung, *Fgf2 *is expressed in alveolar type II cells [49], and may have multiple biological activities *in vitro *and *in vivo*, including angiogenesis, mitogenesis, and cellular differentiation [50]. Additionally, induction of *Fgf2 *expression can influence cell proliferation and biosynthetic events that are important to the proper resolution of tissue injury in the lung [51,52]. Thus, increased *Fgf2 *expression may be an important molecular event in the pathogenesis of nickel-induced acute lung injury.

Taken together, the IBMT method successfully identified several transcripts that were significantly changed at various times throughout the development of nickel-induced acute lung injury in mice that were not identified by the SMT method. These transcripts have been previously investigated in the development of lung injury, and may have biological relevance in our mouse model. The lists of top-ranked genes by IBMT but not SMT, and vice versa, are available as supplemental information.

## Conclusion

IBMT has the strength of balancing two important factors in the analysis of microarray data: the degree of independence of variances relative to the degree of identity (i.e. *t*-tests vs. equal variance assumption), and the relationship between variance and signal intensity. We demonstrated that incorporating information about the dependence of the variance of genes on expression intensity level can improve the efficiency of the Empirical Bayes moderated t-statistics, and that properly estimating the prior degrees of freedom is important in estimating the true proportion of false positives. If a non-intensity-based moderated-T is used, and the variance of low expressed genes is higher than average, then an over-representation of low expressed genes will occur in the top ranked differentially expressed transcripts because their variance estimates will be "shrunk" towards the lower overall variability. This in turn results in a higher rate of falsely implicated genes and makes the interpretation of the results more difficult. Indeed, this trend could be seen in the comparison of genes found to be significant in SMT but not IBMT, or vice versa, in the nickel exposure experiment. SMT identified a large number of relatively low expressed genes (49% < 100 signal level; median expression level = 99), often with unknown function, as being significantly changed compared to IBMT (0% < 100 signal level; median expression level = 357). To our knowledge, IBMT is the first to account for the dependence of gene variance on intensity levels in a completely data-dependent manner, without a need for specification of free parameters by the user, within the empirical Bayes analysis framework. Furthermore, as opposed to Cyber-T [3] and Fox [11], IBMT can properly analyze data from any experimental design setup and array platform, including multiple treatments or time series, Affymetrix chips or two-dye arrays, and experiments with both technical and biological replicates. The prior variance levels are estimated using local regression and the prior degrees of freedom are estimated using a consistent estimator based on the Empirical Bayes approach.

The IBMT method outperformed or performed as well as the simple t-statistic, fold change, SMT, and Fox in simulation studies intended to mimic real microarray data and on real microarray data itself. The improved performance of IBMT on spike-in experiments suggests that the pooling of information across genes, as well as accounting for the relationship between the variances and overall intensities of gene expression measurements, is warranted. The "spike-in" Affymetrix datasets also revealed the need to correctly estimate the prior degrees of freedom for correctly estimating the proportion of false positives. By simply accepting user input for this parameter (as in Cyber-T, and indirectly in Fox), one is at risk of either greatly overestimating or underestimating the true accumulation of false positives. For the "spike-in" experiments, this may explain the poorest estimation of the true false positive rate by Cyber-T and Fox. As our results show, all methods underestimated the proportion of false positives in these Affymetrix spike-in datasets. This may partially be due to the design of these experiments, creating correlations that would not be seen in experimental data, or even unintended real changes. However, correlations among genes and microarrays have been observed in experimental data also, and in this case, the significance statistics may be more accurately calculated using a local fdr procedure with an empirical null distribution, as proposed by Efron [21,53], rather than the Benjamini FDR [23] as applied in this paper. Even if no correlations are expected, Efron's local fdr procedure with the theoretical Normal null may improve accuracy in estimating signficance levels for any chosen analysis method.

Our method was also applied to two experimental dual-channel datasets, a simple knockout versus *wildtype *comparison and a time-series experiment. Analysis of these data indicated that IBMT generated the greatest number of genes involved in GO categories significantly enriched with genes determined to be differentially expressed. Although the biological pathways affected in each experiment can be ascribed with limited certainty, in the time series experiment we examined self-consistency among sampling times. Although affected pathways may change across time, it is reasonable to expect that some should be consistent for at least two or more times. Our analysis showed that IBMT had the highest self-consistency. In addition to the comparison of methods using Gene Ontology, interpretation of the results hinted that biological categories found in the MEF *Ahr*^-/- ^experiment using IBMT were more consistent with functions previously ascribed to this receptor. IBMT also provided a greater percent of genes directly relevant to what is currently known of the response to Nickel exposure in mice.

## Methods

### Mice and exposure protocol

Two dual-channel microarray experiments were performed. The first was a comparison of *wildtype *mouse embryo fibroblast (MEF) cells to aryl-hydrocarbon receptor (*Ahr*) knockout MEF cells. Four biological replicate cell cultures each of *wildtype *and knockout cells were compared, each with dye labelling switched for the second technical replicate of each biological pair.

The second dataset has been published [19] and the methods are summarized here. 129S1/SvImJ strain mice (females, age 7–10 weeks) were purchased from The Jackson Laboratory (Bar Harbor, ME). All mice were housed in our animal facilities ≥ 1 week prior to exposure. Nickel aerosol was generated from 50 mM NiSO_4_•6H_2_O (Sigma, St. Louis, MO) and monitored as described previously [39]. Mice were exposed to 150 ± 15 *μ*g Ni^2+^/m^3 ^in a 0.32-m^3 ^stainless steel inhalation chamber. All experimental protocols were reviewed and approved by the Institutional Animal Care and Use Committee at the University of Cincinnati Medical Center.

Mice were exposed to aerosolized nickel for 3, 8, 24, 48, and 72 h. Following exposure, mice were killed with pentobarbital (followed by exsanguination), and the lungs were removed, placed in liquid nitrogen, and stored at -80°C. Total cellular RNA was isolated from frozen lung tissue with TRIzol (Invitrogen), and quantity was assessed by A260/A280 spectrophotometric absorbance (SmartSpec 3000, Bio-Rad, Hercules, CA). RNA quality was assessed by separation with a denaturing formaldehyde/agarose/ethidium bromide gel, and quantified by analysis with an Agilent Bioanalyzer (Quantum Analytics, Foster City, CA) [19].

### Microarray hybridizations

The two real datasets were performed using Qiagen-Operon's *mus musculus *version 1.1 70-mer oligonucleotide library, representing 13,664 annotated transcripts. The first dataset is a simple comparison of *wildtype *mouse embryo fibroblast (MEF) cells to *Ahr*^-/- ^MEF cells. A similar microarray comparison performed with mouse smooth muscle cells has previously been published [16-18]. The second dataset has been published [19], but we summarize the methods below. RNA quality for both experiments was assessed by separation with a denaturing formaldehyde/agarose/ethidium bromide gel, and quantified by analysis with an Agilent Bioanalyzer (Quantum Analytics, Inc., Foster City, CA). To examine differential gene expression, a 70-mer oligonucleotide library, representing 13,443 mouse genes (Operon Biotechnologies, Inc., Huntsville, AL), was used by the Genomic and Microarray Laboratory, Center for Environmental Genetics, University of Cincinnati,  was used to fabricate microarrays. The microarray hybridisations were carried out as described [16,18]. For the AHR experiment, each biological replicate consisted of one mouse cell culture, and for the Ni-treatment experiment, each exposure group consisted of nine mice. RNA from three mice was pooled for each microarray, and three separate microarrays per exposure group were compared to non-exposed controls. Both experiments were performed using 20 *μ*g total RNA per array. Each sample of mRNA was reverse transcribed and tagged with either fluorescent Cyanine 3 (Cy3) or Cyanine 5 (Cy5) (e.g., Cy3 forcontrol and Cy5 for72-h exposure). Cy3 and Cy5 samples were co-hybridized with the printed 70-mers. Following hybridization, slides were washed and scanned at 635 (Cy5) and 532 (Cy3) nm (GenePix 4000B, Axon Instruments, Inc., Union City, CA).

### Data normalization and analysis

Microarray protocols and analyses were performed as described in [16-18,56]. Briefly, microarray hybridization data representing raw spot intensities generated by the GenePix^® ^Pro v5.0 software and data normalization was performed for each microarray separately. First, channel specific local background intensities were subtracted from the median intensity of each channel (Cy3 and Cy5). Second, background adjusted intensities were log-transformed and the differences (R) and averages (A) of log-transformed values were calculated as R = log_2_(X1) - log_2_(X2) and A = [log_2_(X1) + log_2_(X2)]/2, where X1 and X2 denote the Cy5 and Cy3 intensities after subtracting local backgrounds, respectively. Third, data centering was performed by fitting the array-specific local regression model of R as a function of A [57]. Normalized log-intensities for the two channels were then calculated, and statistical analysis was performed for each gene separately by fitting a mixed effects linear model [58]. For the MEF *Ahr*^-/- ^experiment the model used was: Y_ijkl _= *μ *+ A_i _+ S_j _+ M(S)_kj _+ C_l _+ 'Ω_ijkl_, where Y_ijkl _corresponds to the normalized log-intensity on the i^th ^array (i = 1,..., 8), with the j^th ^treatment (j = 1, 2), for the k^th ^mouse, and labeled with the *l*^th ^dye (*l *= 1 for Cy5, and 2 for Cy3). *μ *is the overall mean log-intensity, A_i _is the effect of the i^th ^array, S_j _is the effect of the j^th ^treatment, M(S)_kj _is the effect of the k^th ^mouse with treatment j, and C_k _is the effect of the k^th ^dye. Assumptions about the model parameters were the same as described elsewhere [58], with array and mouse effects assumed to be random, and treatment, and dye effects assumed to be fixed. The model for the second dataset was as described above, with the exception of no mouse-within-treatment effect, and a higher number of arrays (5·3 = 15) and treatment conditions (6) [19]. Ordinary T-statistics and estimates of fold change were calculated for each gene using this model. The SMT [12] and IBMT significance levels were then calculated as described above.

## Availability and requirements

We have implemented IBMT as an R function [54] which can be downloaded as a text file along with all other supplemental material from our supporting website [55] or from the supplemental material [see Additional file 2]. The function requires R statistical software and is most easily implemented using the functionality of the *limma *package [12], but can also be used in conjunction with other linear model or mixed model analyses.

## Authors' contributions

MAS conceived of and developed the specific methodology, and drafted the manuscript. MM participated in the conception of this methodology and provided guidance in the development, design, and drafting of the manuscript, and SS contributed to the statistical details of the method. CRT, SCW, and GDL provided interpretation of the biological results from the dual-channel datasets, and CRT additionally oversaw the microarray hybridizations for the two dual-channel experiments.

## Supplementary Material

Additional file 1 **Supplemental Material**. PDF file containing several additional figures and tables. Click here for file

Additional file 2 **IBMT R-code**. Text file containing R-function for implementing IBMT with the *limma *Bioconductor package or other linear or mixed model analysis. Click here for file
