# Supplementary material for: Intensity-based hierarchical Bayes method improves testing for differentially expressed genes in microarray experiments
Source: BMC Bioinformatics. 2006 Dec 19;7:538. doi: 10.1186/1471-2105-7-538 (PMC1781470; doi:10.1186/1471-2105-7-538)

Supplemental Information

Intensity-based hierarchical Bayes method improves testing for differentially expressed genes in microarray experiments

Maureen A Sartor, Craig R Tomlinson , Scott C Wesselkamper, Siva Sivaganesan, George D Leikauf, Mario Medvedovic§

§Corresponding author: mario.medvedovic@uc.edu

Outline for Supplemental Material:

1) Dependency of log-variance on log-expression intensity in simulation study
2) Control of false positive rate in simulation study for additional parameter sets
3) Improved relative performance of t-test with higher sample degrees of freedom
4) Control of false positive rate in Affymetrix "spike-in" dataset
5) Full list of significant Gene Ontology categories for MEF *Ahr*$^{-/-}$ dataset
6) Top ranked genes from each of 4 methods for MEF *Ahr*$^{-/-}$ dataset
7) List of significant Gene Ontology categories for Nickel time course
8) Top ranked genes in IBMT, but not SMT, and vice versa for Nickel data
9) Variance-Intensity relationship for latin-square experiment
10) Robustness of method to *loess* span parameter, from latin-square experiment

1) Dependency of log-variance on log-expression intensity in simulation study

Figure S1: Example of Local regression estimation of log-variance. Similar results were found using other parameters conditions.

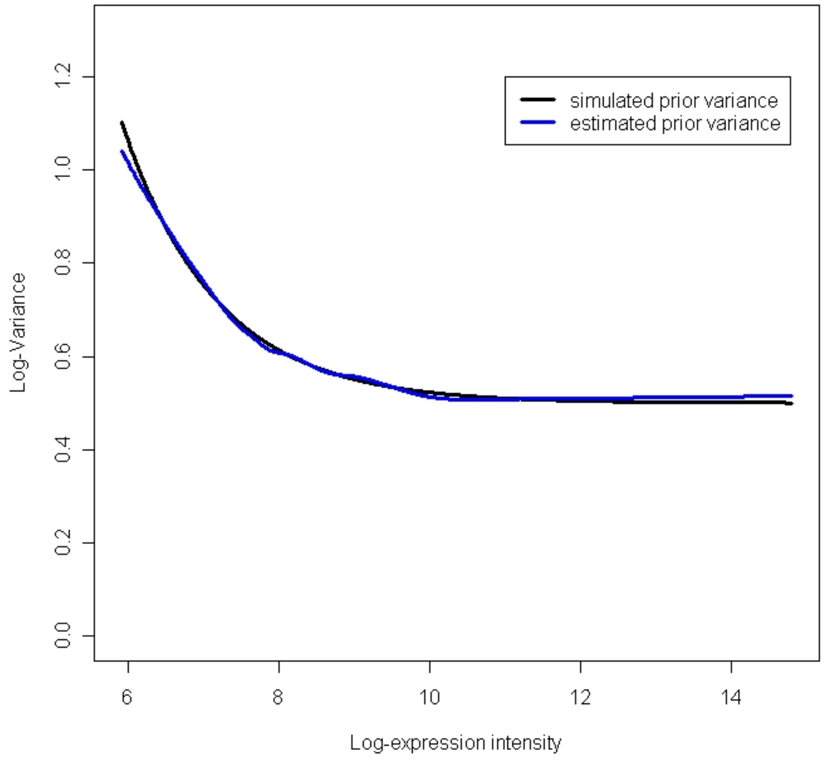

2) **Control of False Positive Rate in simulations studies for additional parameter sets**

Figure S2: Actual vs. estimated false positive rates were plotted for a sample of parameter sets in the simulations described. 300 genes were simulated as differentially expressed, and results shown are the average of 100 simulations. Similar results, showing first 3 methods (t-test, SMT, and IBMT) correctly controlled the false positive rate, were obtained using all other parameter sets.

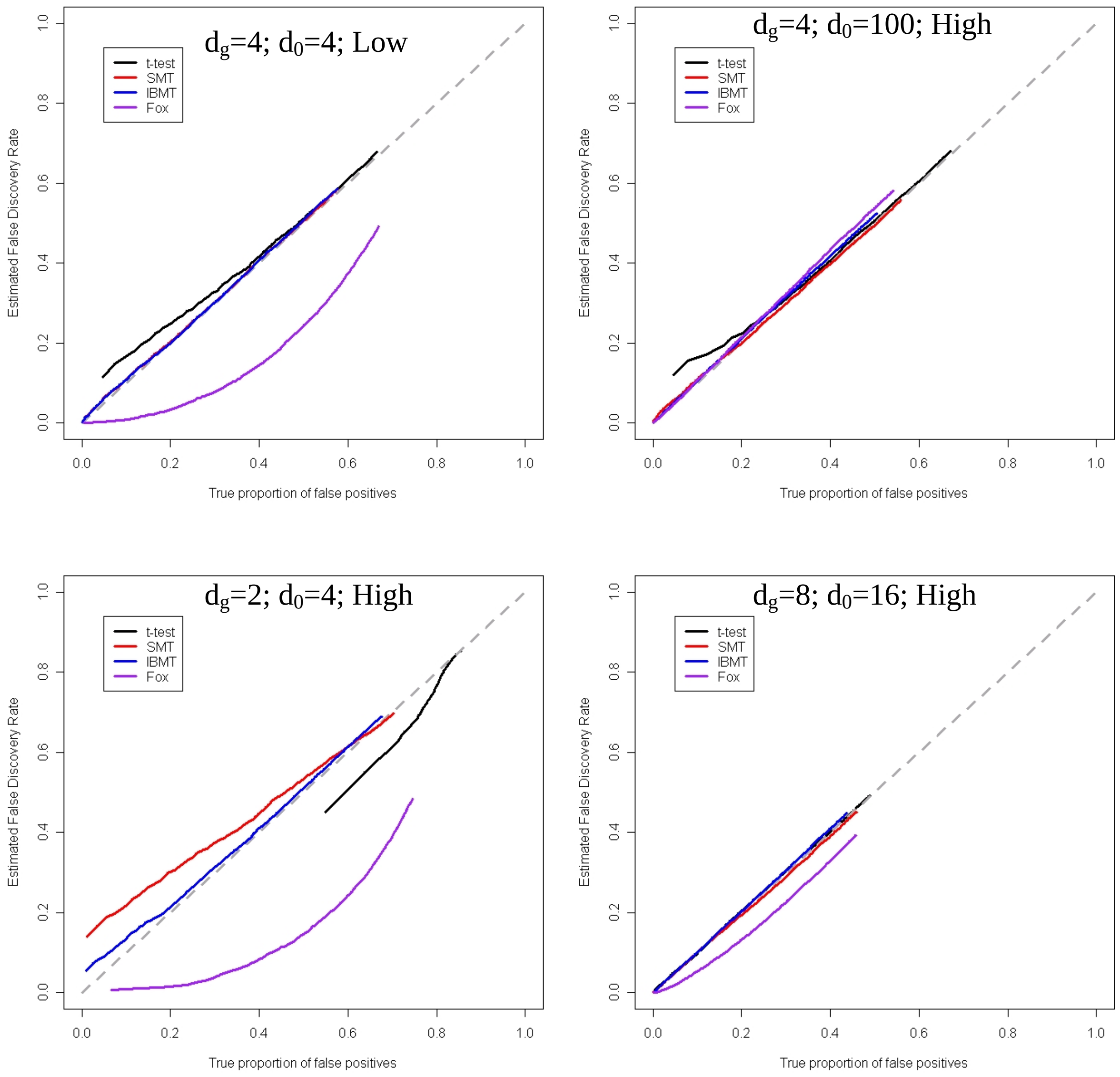

3) **Improved relative performance of t-test with higher sample degrees of freedom**

Figure S3: Accumulation of false positives by increasing number of genes determined to be significant for the simple t-test (black), SMT (red), and IBMT (blue). Sample degrees of freedom varies from A) 4, B) 8, C) 12, and D) 16, which prior degrees of freedom remains constant at 16. The simple t-test improves as the sample degrees of freedom increases relative to SMT and IBMT.

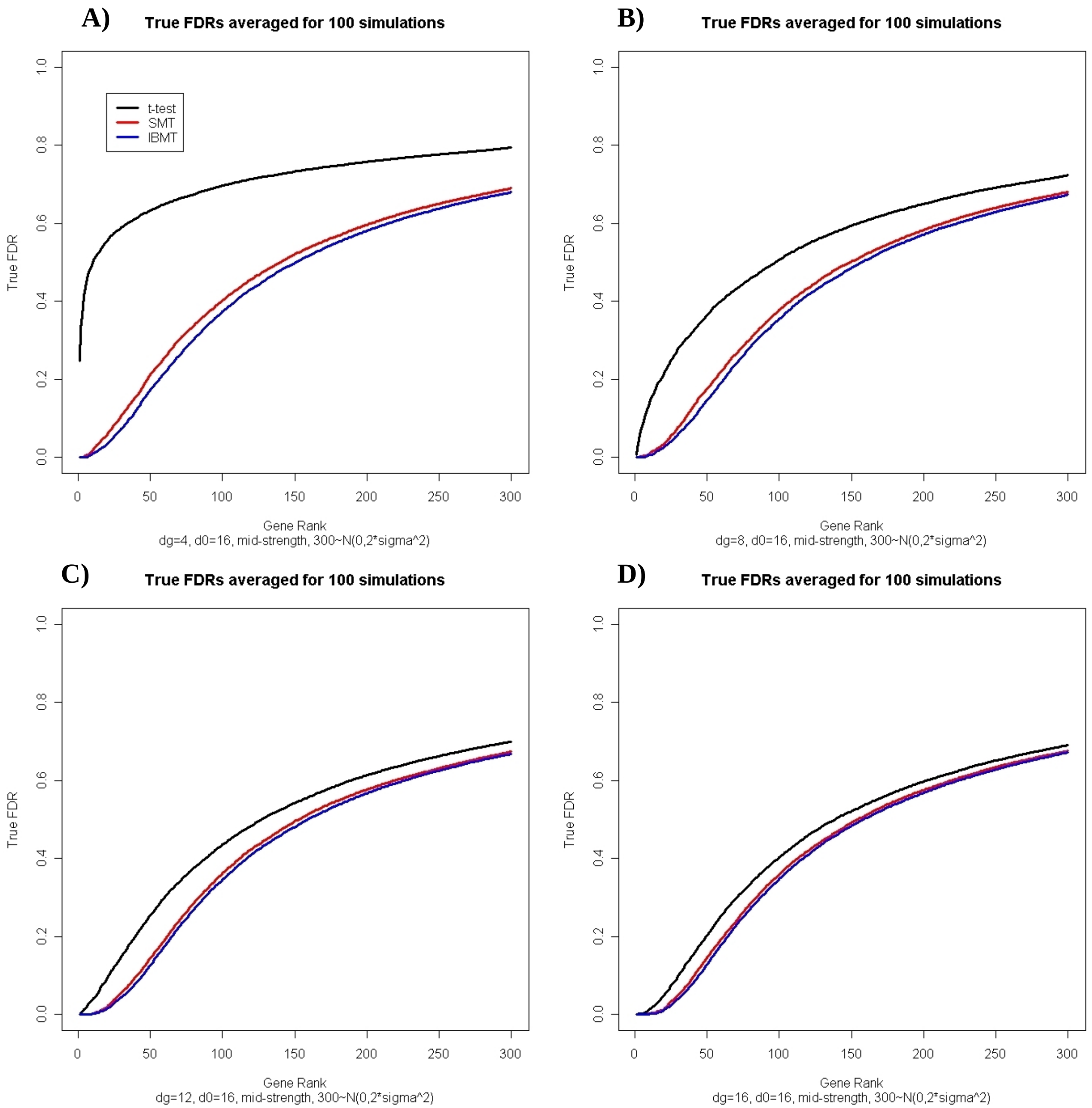

4) Control of false positive rate in Affymetrix "spike-in" dataset

Table S2: Comparison of methods to control false positive rate at the 1% and 5% levels. The first column lists the cutoff value used for each row. The second column displays the number of genes that should be determined significant using the corresponding cutoff from the first column, if the false positive rate is correctly controlled. The 3rd column shows number of genes estimated to be significant using the Benjamini-Hochberg FDR corresponding cutoff. The number and percent of "extra" genes determined to be significant, beyond what would be found according to the true q-value, is indicated in the last column. For both cutoffs, IBMT find the lowest percent of additional genes.

	x(q-value cutoff)	# genes with true q-value<*x*	# genes with estimated FDR<*x*	# (%) genes deviation
IBMT	0.05	926	1555	629 (40%)
Cyber-T	0.05	871	1624	753 (46%)
SMT	0.05	708	1517	809 (53%)
t-test	0.05	737	1309	572 (44%)
IBMT	0.01	659	1216	557 (46%)
Cyber-T	0.01	617	1363	746 (55%)
SMT	0.01	344	1179	835 (71%)
t-test	0.01	253	931	678 (73%)

5) **Full list of significant Gene Ontology categories for MEF *Ahr*$^{-/-}$ dataset**

Table S2: Gene Ontology categories that had a bonferroni-adjusted p-value<0.10 for each of the 4 tested methods, listing category (# significant genes in category), and Bonferroni-adjusted *p*-value.

GO category rank	T-test	FOLD	SMT (eBayes)	IBMT
1	Extracellular space (77) 5.3E-003	Extracellular (91): 2.6E-005	Extracellular (90) 5.9E-005	Extracellular (92) 1.8E-006
2	Extracellular (84) 9.1E-003	Extracellular space (82) 6.25E-005	Extracellular space (81) 1.4E-004	Response to biotic stimulus (39) 1.0E-005
3	Integrin binding (5) 2.8E-002	Signal transducer activity (67) 1.7E-002)	Receptor binding (27) 1.2E-003	Extracellular space (80) 6.7E-005
4	Spermidine biosynthesis (3) 4.1E-002	Organogenesis (38) 3.5E-002	Chemoattractant activity (8) 3.6E-003	Response to external stimulus (46) 2.7E-004
5	Spermine biosynthesis (3) 4.1E-002	Chemoattractant activity (7) 4.3E-002	Signal transducer activity (68) 4.0E-003	Defense response (34) 2.9E-004
6	Carboxy peptidase activity (6) 6.8E-002	Receptor binding (24) 4.9E-002	Response to biotic stimulus (33) 8.6E-003	Signal transducer activity (68) 2.0E-003
7		Histogenesis and organogenesis (9) 7.8E-002	Chemokine receptor binding (7) 1.9E-002	Chemoattractant activity (8) 3.1E-003
8		Morphogenesis (39) 8.6E-002	Chemokine activity (7) 1.9E-002	Immune response (27) 6.1E-003
9			Integrin binding (5) 1.9E-002	Response to pest/pathogen/parasite (19) 9.8E-003
10			G-protein-coupled receptor binding (7) 2.4E-002	Chemokine activity (7) 1.6E-002
11			Spermidine biosynthesis 3.2E-002	Chemokine receptor binding (7) 1.6E-002
12			Spermine biosynthesis (3) 3.2E-002	G-protein-coupled receptor binding (7) 2.1E-002
13			Defense response (29) 6.1E-002	Spermine biosynthesis (3) 3.0E-002
14				Spermidine biosynthesis (3) 3.0E-002
15				Extracellular matrix (18) 3.0E-002
16				Receptor binding (23) 7.4E-002
17				Response to stress (29) 8.4E-002

6) Top ranked genes from each of 4 methods for MEF *Ahr*$^{-/-}$ dataset

Table S2: Top ranked genes from each of 4 methods. For each gene, the Entrez Gene ID: Gene Symbol (Average expression level) and fold change are shown. Positive fold change values indicate higher expression in *Ahr*$^{-/-}$ cells.

Rank	T-test	SMT	IBMT	FOLD
1	13386: Dlk1 (876) 16.23	13386: Dlk1 (876) 16.23	16002: Igf2 (1296) 15.38	20379: Sfrp4(117) 24.97
2	13217: Defcr-ps1 (196) -3.74	16002: Igf2 (1296) 15.38	13386: Dlk1 (876) 16.23	16012: Igfbp6(348) 24.46
3	21985: Tpd52 (636) -4.24	69137: 2200002K05Rik (2416) -10.24	16012: Igfbp6 (348) 24.46	15427: Hoxc9(116) 17.71
4	69137: 2200002K05Rik (2416) -10.24	69065: Chac1 (492) -14.31	69137: 2200002K05Rik (2416) -10.24	13386: Dlk1(876) 16.23
5	15937: Ier3 (5828) -3.97	15426: Hoxc8 (383) 10.53	69065: Chac1 (492) -14.31	16002: Igf2(1296) 15.38
6	20198: S100a4 (1143) 3.11	57349: Cxcl7 (182) -12.85	53606: G1p2 (830) 7.69	22441: Xlr(45) -15.13
7	23918: Impdh2 (2468) -2.13	677168: LOC677168 (639) 8.36	677168: LOC677168 (639) 8.36	69065: Chac1(492) -14.31
8	21927: Tnfaip1 (850) -1.58	12484: Cd24a (639) -6.94	15426: Hoxc8 (383) 10.53	57349: Cxcl7(182) -12.85
9	16002: Igf2 (1296) 15.38	53606: G1p2 (830) 7.69	20715: Serpina3g (354) 11.14	77125: 9230117N10Rik(162) 12.12
10	20363: Sepp1 (1036) 3.06	16012: Igfbp6 (348) 24.46	26421: Mrpplf3 (407) -9.16	20715: Serpina3g(354) 11.14
11	12484: Cd24a (639) -6.94	15427: Hoxc9 (116) 17.71	20319: Sfrp2 (1690) 5.63	15426: Hoxc8(383) 10.53
12	15426: Hoxc8 (383) 10.53	20319: Sfrp2 (1690) 5.63	12484: Cd24a (639) -6.94	20728: Spic(83) 10.45
13	69065: Chac1 (492) -14.31	12797: Cnn1 (1745) -5.35	12797: Cnn1 (1745) -5.35	69137: 2200002K05Rik(2416) -10.24
14	12797: Cnn1 (1745) -5.35	20847: Stat2 (248) 6	13179: Dcn (2875) 4.8	13115: Cyp27b1(111) 10.08
15	17750: Mt2 (2416) 3.11	56089: Ramp3 (204) -5.43	21826: Thbs2 (1566) 5.15	12628: Cfh(227) 9.6
16	20319: Sfrp2 (1690) 5.63	18812: Plf2 (410) -5.19	20379: Sfrp4 (117) 24.97	26421: Mrpplf3(407) -9.16
17	18812: Plf2 (410) -5.19	21985: Tpd52 (636) -4.24	12258: Serping1 (3465) 4.52	20304: Ccl5(91) 9
18	15402: Hoxa5 (178) 3.76	26421: Mrpplf3 (407) -9.16	57349: Cxcl7 (182) -12.85	677168: LOC677168(639) 8.36
19	56089: Ramp3 (204) -5.43	15937: Ier3 (5828) -3.97	12825: Col3a1 (4622) 4.31	80861: D11Lgp2e(109) 8.02
20	677168: LOC677168 (639) 8.36	13179: Dcn (2875) 4.8	12628: Cfh (227) 9.6	12628: Cfh(96) 7.7
21	17313: Mglap (4742) 4.1	15284: Hlx (378) 6.63	18812: Plf2 (649) -5.41	53606: G1p2(830) 7.69
22	54427: Dnmt3l (111) -1.33	20379: Sfrp4 (117) 24.97	15284: Hlx (378) 6.63	15425: Hoxc6(46) 7.66
23	18787: Serpine1 (4486) -3.11	20666: Sox11 (63) -5.92	17313: Mglap (4742) 4.1	53622: Krt2-18(62) -7.61
24	66054: Cndp2 (437) -2.38	13217: Defcr-ps1 (196) -3.74	17294: Mest (446) 5.99	54123: Irf7(269) 7.51
25	11640: Akap1 (318) -1.34	22361: Vnn1 (194) -4.88	77125: 9230117N10Rik (162) 12.12	78600: Pde6h(61) 7.34
26	57349: Cxcl7 (182) -12.85	17313: Mglap (4742) 4.1	20750: Spp1 (1597) 4.22	27062: Cadps(40) -7.18
27	20847: Stat2 (248) 6	12759: Clu (467) 5.04	15937: Ier3 (5828) -3.97	12484: Cd24a(639) -6.94
28	77134: Hnrpa0 (6038) 2.21	12258: Serping1 (3465) 4.52	54123: Irf7 (269) 7.51	56504: Stk23(79) 6.9
29	11946: Atp5a1 (6106) -1.52	15402: Hoxa5 (178) 3.76	15427: Hoxc9 (116) 17.71	68797: Pdgfrl(197) 6.8
30	13179: Dcn (2875) 4.8	12825: Col3a1 (4622) 4.31	14219: Ctgf (2372) -3.88	15284: Hlx(378) 6.63
31	53606: G1p2 (830) 7.69	99543: Olfml3 (675) 4.12	12759: Clu (467) 5.04	192216: Tm4sf10(69) -6.61
32	18242: Oat (1608) -1.68	54123: Irf7 (269) 7.51	21384: Tbx15 (584) -4.58	20310: Cxcl2(117) 6.48
33	58916: Ttid (64) -1.65	20609: Sstr5 (151) 4.12	18812: Plf2 (410) -5.19	22678: Zfp2(87) 6.28
34	76983: Scfd1 (1729) -1.33	20750: Spp1 (1597) 4.22	12931: Crlf1 (494) -4.65	20847: Stat2(248) 6

35	68265: Iqcf3 (966) 2.45	80861: D11Lgp2e (109) 8.02	21985: Tpd52 (636) -4.24	17294: Mest(446) 5.99
36	110253: Triobp (1191) 1.91	21826: Thbs2 (1566) 5.15	99543: Olfml3 (675) 4.12	15959: Ifit3(190) 5.94
37	78267: Klhdc8b (944) 2.51	67775: Rtp4 (190) 5.85	13096: Cyp2c37 (869) 3.88	20666: Sox11(63) -5.92
38	68836: Mrpl52 (968) -2	13610: Edg3 (617) 3.85	79565: Wbscr27 (1959) 3.45	20716: Serpina3n(84) 5.9
39	19401: Rara (279) 2.69	20198: S100a4 (1143) 3.11	14871: Gstt1 (574) 4.16	67775: Rtp4(190) 5.85
40	22361: Vnn1 (194) -4.88	72174: Atxn7l4 (475) 3.96	20847: Stat2 (248) 6	20319: Sfrp2(1690) 5.63
41	11702: Amd1 (592) -2.4	20716: Serpina3n (84) 5.9	68797: Pdgfrl (197) 6.8	56089: Ramp3(204) -5.43
42	99543: Olfml3 (675) 4.12	12628: Cfh (227) 9.6	23892: Cktsf1b1 (344) 4.82	18812: Plf2(649) -5.41
43	11792: Apex1 (680) -1.8	20363: Sepp1 (1036) 3.06	13610: Edg3 (617) 3.85	15377: Foxa3(36) -5.39
44	13885: Es10 (1973) -2.35	17750: Mt2 (2416) 3.11	18787: Serpine1 (4486) -3.11	76933: Ifi27(153) 5.38
45	12010: B2m (9394) 2.17	18787: Serpine1 (4486) -3.11	14264: Fmod (420) 4.34	16529: Kcnk5(43) -5.38
46	18102: Nme1 (3869) -1.4	12836: Col7a1 (375) -3.55	20296: Ccl2 (3593) 3.08	12797: Cnn1(1745) -5.35
47	20315: Cxcl12 (1205) 3.12	14219: Ctgf (2372) -3.88	17750: Mt2 (2416) 3.11	18812: Plf2(410) -5.19
48	21417: Zfhx1a (322) -2.3	14871: Gstt1 (574) 4.16	12642: Ch25h (681) 3.6	21826: Thbs2(1566) 5.15
49	11843: Arf4 (1395) -1.68	27528: D0H4S114 (263) 3.82	72174: Atxn7l4 (475) 3.96	12759: Clu(467) 5.04
50	12825: Col3a1 (4622) 4.31	26941: Slc9a3r1 (989) 3.33	26941: Slc9a3r1 (989) 3.33	112407: Egln3(152) 5.03
51	20609: Sstr5 (151) 4.12	20315: Cxcl12 (1205) 3.12	13115: Cyp27b1 (111) 10.08	22361: Vnn1(194) -4.88
52	15439: Hp (2965) 1.95	11475: Acta2 (336) -3.68	15959: Ifit3 (190) 5.94	23892: Cktsf1b1(344) 4.82
53	12793: Cnih (1040) -2.07	23892: Cktsf1b1 (344) 4.82	67775: Rtp4 (190) 5.85	13179: Dcn(2875) 4.8
54	12579: Cdkn2b (731) 2.23	77125: 9230117N10Rik (162) 12.12	20315: Cxcl12 (1205) 3.12	14570: Arhgdig(79) -4.73
55	15032: H2-T17 (1305) 1.82	79565: Wbscr27 (1959) 3.45	20198: S100a4 (1143) 3.11	12931: Crlf1(494) -4.65
56	69219: Ddah1 (626) -1.75	73690: Glipr1 (739) -3.24	56089: Ramp3 (204) -5.43	21384: Tbx15(584) -4.58
57	23876: Fbln5 (1186) 2.09	56338: Txnip (366) 3.3	73690: Glipr1 (739) -3.24	22157: Tulp1(71) 4.57
58	12258: Serping1 (3465) 4.52	22634: Plagl1 (201) 4.12	20363: Sepp1 (1036) 3.06	12258: Serping1(3465) 4.52
59	67105: 1700034H14Rik (320) -1.59	21645: Tcte1 (655) -3.17	53761: Bat2 (630) 3.26	258430: Olfr938(126) 4.48
60	16007: Cyr61 (5643) -1.7	13732: Emp3 (72) 3.13	21645: Tcte1 (655) -3.17	14264: Fmod(420) 4.34
61	16008: Igfbp2 (384) -2.26	20296: Ccl2 (3593) 3.08	13400: Dm15 (310) 3.99	12825: Col3a1(4622) 4.31
62	15039: H2-T22 (1535) 1.99	16950: Loxl3 (543) 3.2	20392: Sgce (359) 3.75	21985: Tpd52(636) -4.24
63	14085: Fah (942) -1.45	12931: Crlf1 (494) -4.65	17840: Mup1 (979) 2.93	20750: Spp1(1597) 4.22
64	11998: Avp (135) 1.91	258430: Olfr938 (126) 4.48	13874: Ereg (583) -3.19	15415: Hoxb7(46) -4.17
65	18111: Nnat (109) 2.61	16399: Itga2b (177) 3.42	21893: Tlm (2271) 2.67	14871: Gstt1(574) 4.16
66	59028: Rcl1 (333) -1.6	13096: Cyp2c37 (869) 3.88	20393: Sgk (1758) -2.72	22634: Plagl1(201) 4.12
67	20666: Sox11 (63) -5.92	17840: Mup1 (979) 2.93	16950: Loxl3 (543) 3.2	20609: Sstr5(151) 4.12
68	17035: Lxn (2296) 2.53	20304: Ccl5 (91) 9	18811: Plf (1863) -2.69	99543: Olfml3(675) 4.12
69	286940: Flnb (891) -2.55	19401: Rara (279) 2.69	15404: Hoxa7 (585) 3.15	17313: Mglap(4742) 4.1
70	14964: H2-D1 (967) 1.83	13874: Ereg (583) -3.19	80861: D11Lgp2e (109) 8.02	16433: Itmap1(205) 4.05
71	13610: Edg3 (617) 3.85	20765: Sprr2k (109) 3.4	22361: Vnn1 (194) -4.88	18181: Nrf1(53) -4.01
72	67963: Npc2 (378) 2.73	67963: Npc2 (378) 2.73	12836: Col7a1 (375) -3.55	13400: Dm15(310) 3.99
73	23997: Psmd13 (632) -1.33	67993: Nudt12 (383) 2.73	11475: Acta2 (336) -3.68	20342: Selenbp2(68) 3.99
74	67993: Nudt12 (383) 2.73	67286: Rabl5 (490) 2.88	74558: Gvin1 (291) 3.88	15937: Ier3(5828) -3.97
75	26941: Slc9a3r1 (989) 3.33	64817: Svep1 (738) 2.79	23962: Oasl2 (279) 3.94	72174: Atxn7l4(475) 3.96
76	30926: Txnl2 (1393) -1.55	20728: Spic (83) 10.45	22644: Rnf103 (4139) -2.55	23962: Oasl2(279) 3.94
77	14866: Gstm5 (826) -1.26	20393: Sgk (1758) -2.72	11532: Adh5 (821) 2.87	16633: Klra2(49) 3.91
78	20393: Sgk (1758) -2.72	18727: Pira4 (460) 3.16	19242: Ptn (506) 3.18	12269: C4bp(56) 3.89
79	118454: Gja12 (1285) -2.33	20342: Selenbp2 (68) 3.99	13711: Elf5 (298) -3.81	14219: Ctgf(2372) -3.88

80	20750: Spp1 (1597) 4.22	18301: Fxyd5 (269) -2.9	NM_014195: L1Md-Tf29 (505) 3.15	74558: Gvin1(291) 3.88
81	12759: Clu (467) 5.04	18111: Nnat (109) 2.61	17318: Mid1 (520) 3.13	13096: Cyp2c37(869) 3.88
82	20657: Sod3 (1255) 1.84	19242: Ptn (506) 3.18	76933: Ifi27 (153) 5.38	13610: Edg3(617) 3.85
83	16336: Insl3 (135) 1.55	78267: Klhdc8b (944) 2.51	20728: Spic (83) 10.45	27528: D0H4S114(263) 3.82
84	15284: Hlx (378) 6.63	68797: Pdgfrl (197) 6.8	17035: Lxn (2296) 2.53	13711: Elf5(298) -3.81
85	19128: Pros1 (343) 1.51	26419: Mapk8 (475) 2.81	18727: Pira4 (460) 3.16	15227: Foxf1a(183) -3.79
86	15507: Hspb1 (1951) -1.59	66175: Mustn1 (496) 2.72	20304: Ccl5 (91) 9	99899: Ifi44(115) 3.79
87	16323: Inhba (454) -2.01	15959: Ifit3 (190) 5.94	27528: D0H4S114 (263) 3.82	15402: Hoxa5(178) 3.76
88	14237: Foxd4 (323) -1.81	192216: Tm4sf10 (69) -6.61	64817: Svep1 (738) 2.79	20392: Sgce(359) 3.75
89	12836: Col7a1 (375) -3.55	286940: Flnb (891) -2.55	20310: Cxcl2 (117) 6.48	13217: Defcr-ps1(196) -3.74
90	15427: Hoxc9 (116) 17.71	14451: Gas1 (852) 2.72	14451: Gas1 (852) 2.72	21788: Tfpi(82) -3.72
91	26407: Map3k4 (191) -1.22	16633: Klra2 (49) 3.91	56338: Txnip (366) 3.3	11475: Acta2(336) -3.68
92	67738: Ppid (2054) -1.86	17035: Lxn (2296) 2.53	112407: Egln3 (152) 5.03	54526: Syt10(112) -3.68
93	15115: Hars (850) -1.9	68265: Iqcf3 (966) 2.45	12628: Cfh (96) 7.7	17450: Morc(100) 3.62
94	16012: Igfbp6 (348) 24.46	80285: Parp9 (242) 3.29	73353: Arpm2 (668) -2.77	12642: Ch25h(681) 3.6
95	67834: Idh3a (1504) -1.94	66054: Cndp2 (437) -2.38	20364: Sepw1 (1861) 2.46	26410: Map3k8(207) 3.57
96	171095: Il17rc (123) -2.53	171095: Il17rc (123) -2.53	21928: Tnfaip2 (691) 2.73	12836: Col7a1(375) -3.55
97	17427: Mns1 (168) -1.67	20607: Sstr3 (362) 2.67	22634: Plagl1 (201) 4.12	23792: Adam23(99) 3.53
98	66413: Psmd6 (1642) -1.77	16627: Klra1 (443) -2.65	16433: Itmap1 (205) 4.05	79565: Wbscr27(1959) 3.45
99	13202: Ddt (800) 2.24	21928: Tnfaip2 (691) 2.73	16737: L1Md-Tf5 (1009) 2.56	16399: Itga2b(177) 3.42
100	12579: Cdkn2b (1000) 2.31	11702: Amd1 (592) -2.4	14118: Fbn1 (908) 2.59	20765: Sprr2k(109) 3.4
101	64817: Svep1 (738) 2.79	212706: C330016O10Rik (103) 2.95	56791: Ubce8 (300) 3.37	54138: Atxn10(49) 3.4
102	18013: Neurod2 (565) -1.52	19735: Rgs2 (108) 2.53	52668: D12Ertd647e (1749) 2.42	11816: Apoe(216) 3.4
103	26421: Mrpplf3 (407) -9.16	14264: Fmod (420) 4.34	67286: Rabl5 (490) 2.88	58185: Rsad2(103) 3.38
104	72174: Atxn7l4 (475) 3.96	18049: Ngfb (337) -2.6	14456: Gas6 (714) 2.63	27081: Zfp275(131) 3.38
105	21810: Tgfbi (237) -2.21	66447: Mgst3 (280) 2.68	16734: L1Md-Tf26 (1023) 2.51	56791: Ubce8(300) 3.37
106	58520: ORF11 (769) 1.86	20364: Sepw1 (1861) 2.46	286940: Flnb (891) -2.55	79361: Stx1bl(55) 3.37
107	72654: Ccdc12 (668) -1.99	13885: Es10 (1973) -2.35	13885: Es10 (1973) -2.35	18616: Peg3(141) -3.35
108	13732: Emp3 (72) 3.13	74359: 4931414P19Rik (309) 2.57	78267: Klhdc8b (944) 2.51	16367: Irs1(80) 3.35
109	20187: Ryk (1612) -1.74	24059: Slco2a1 (341) 2.86	18214: Ddr2 (1385) 2.4	26941: Slc9a3r1(989) 3.33
110	73690: Glipr1 (739) -3.24	26921: Map4k4 (465) 2.5	26419: Mapk8 (475) 2.81	19878: Rock2(243) 3.31
111	20296: Ccl2 (3593) 3.08	11816: Apoe (216) 3.4	16736: L1Md-Tf30 (1021) 2.46	56338: Txnip(366) 3.3
112	20364: Sepw1 (1861) 2.46	14824: Grn (592) 2.45	16819: Lcn2 (1153) 2.42	80285: Parp9(242) 3.29
113	117109: Pop5 (208) 1.86	16819: Lcn2 (1153) 2.42	68265: Iqcf3 (966) 2.45	66695: Aspn(155) 3.29
114	21645: Tcte1 (655) -3.17	53761: Bat2 (630) 3.26	107765: Ankrd1 (1757) -2.31	56760: Clec1b(120) 3.26
115	70316: Ndufab1 (1441) -1.44	21417: Zfhx1a (322) -2.3	66175: Mustn1 (496) 2.72	53761: Bat2(630) 3.26
116	93838: Dqx1 (411) -1.57	26410: Map3k8 (207) 3.57	68252: A030007L17Rik (453) 2.78	73690: Glipr1(739) -3.24
117	16819: Lcn2 (1153) 2.42	14238: Foxf2 (132) -2.92	21384: Tbx15 (1237) -2.37	11639: Ak4(81) -3.24
118	15006: H2-Q1 (573) 2.29	27423: Klra15 (86) -2.65	26367: Ceacam2 (14279) 2.27	16950: Loxl3(543) 3.2
119	17840: Mup1 (979) 2.93	12628: Cfh (96) 7.7	12982: Csf2ra (7976) 2.25	13874: Ereg(583) -3.19
120	13591: Ebf1 (269) 1.95	27056: Irf5 (101) -2.67	13217: Defcr-ps1 (196) -3.74	11803: Aplp1(76) -3.19

121	74400: 4933405K07Rik (152) 2.22	118454: Gja12 (1285) -2.33	16732: L1Md-Tf18 (701) 2.51	19242: Ptn(506) 3.18
122	19063: Ppt1 (926) 2.23	14118: Fbn1 (908) 2.59	97122: Hist2h4 (1102) 2.37	21645: Tcte1(655) -3.17
123	19025: Ppgb (1617) 2.06	67893: Tmem86a (305) 2.83	118454: Gja12 (1285) -2.33	13614: Edn1(133) 3.17
124	56338: Txnip (366) 3.3	260409: Cdc42ep3 (503) 2.56	77134: Hnrpa0 (6038) 2.21	18727: Pira4(460) 3.16
125	56264: Cpxm1 (271) 2.09	16433: Itmap1 (205) 4.05	26410: Map3k8 (207) 3.57	NM_014195: L1Md-Tf29(505) 3.15
126	54613: Siat10 (504) 1.95	12579: Cdkn2b (1000) 2.31	15227: Foxf1a (183) -3.79	15404: Hoxa7(585) 3.15
127	12339: Capn7 (284) -1.5	77134: Hnrpa0 (6038) 2.21	19878: Rock2 (243) 3.31	13732: Emp3(72) 3.13
128	21461: Tcp10b (73) -2.33	56504: Stk23 (79) 6.9	97122: Hist2h4 (1607) 2.26	17318: Mid1(520) 3.13
129	19228: Pthr1 (533) -1.39	20846: Stat1 (317) 2.94	15200: Dtr (368) -2.85	19883: Rora(147) 3.12
130	19735: Rgs2 (108) 2.53	16008: Igfbp2 (384) -2.26	21923: Tnc (1616) -2.26	20315: Cxcl12(1205) 3.12
131	67286: Rabl5 (490) 2.88	16367: Irs1 (80) 3.35	80285: Parp9 (242) 3.29	20198: S100a4(1143) 3.11
132	85308: 1500005A01Rik (2553) 1.69	17294: Mest (446) 5.99	20944: Svs5 (1528) -2.26	18787: Serpine1(4486) -3.11
133	20641: Snrpd1 (707) -1.58	24110: Usp18 (242) 2.99	56418: Ykt6 (881) -2.38	17750: Mt2(2416) 3.11
134	66175: Mustn1 (496) 2.72	21461: Tcp10b (73) -2.33	16738: L1Md-Tf6 (548) 2.56	22598: Xtrp2(30) -3.1
135	78514: Arhgap10 (244) -1.51	12579: Cdkn2b (731) 2.23	16019: Igh-6 (1713) -2.23	54352: Irx5(108) -3.1
136	14824: Grn (592) 2.45	15006: H2-Q1 (573) 2.29	15402: Hoxa5 (178) 3.76	14432: Gap43(225) -3.09
137	64652: Nisch (2494) -1.55	12890: Cplx2 (435) 2.55	20846: Stat1 (317) 2.94	16443: Itsn(75) -3.08
138	72147: Btbd4 (833) -1.65	23918: Impdh2 (2468) -2.13	11816: Apoe (216) 3.4	20296: Ccl2(3593) 3.08
139	54616: Extl3 (330) -1.65	19878: Rock2 (243) 3.31	12424: Cck (478) 2.62	71584: Gdpd2(174) 3.07
140	16950: Loxl3 (543) 3.2	13025: Ctla2b (173) 2.33	20609: Sstr5 (151) 4.12	29848: Olfr158(38) -3.06
141	66406: Sac3d1 (525) -2.24	56418: Ykt6 (881) -2.38	24059: Slco2a1 (341) 2.86	20363: Sepp1(1036) 3.06
142	258604: Olfr970 (368) -2.03	13202: Ddt (800) 2.24	12010: B2m (9394) 2.17	18131: Notch3(62) 3.05
143	79565: Wbscr27 (1959) 3.45	56791: Ubce8 (300) 3.37	22240: Dpysl3 (751) -2.39	17380: Mme(53) -3.03
144	18481: Pak3 (5012) -1.44	12457: Ccrn4l (373) -2.69	16627: Klra1 (443) -2.65	20378: Frzb(237) 3.01
145	22004: Tpm2 (418) -1.89	326619: Hist1h4a (77) -2.91	67938: Mylc2b (1893) 2.19	20308: Ccl9(203) 3
146	56075: Pdss1 (598) -1.88	16740: L1Md-Tf9 (452) 2.5	21825: Thbs1 (4229) -2.14	24110: Usp18(242) 2.99
147	18655: Pgk1 (1657) -1.89	12010: B2m (9394) 2.17	12579: Cdkn2b (1000) 2.31	57429: Sultx1(86) 2.97
148	14219: Ctgf (2372) -3.88	214682: Myo3a (88) 2.47	19288: Ptx3 (5948) -2.15	14160: Gpr49(25) 2.95
149	27528: D0H4S114 (263) 3.82	56532: Ripk3 (215) -2.55	260409: Cdc42ep3 (503) 2.56	212706: C330016O10Rik(103) 2.95
150	53378: Sdcbp (331) -1.61	19063: Ppt1 (926) 2.23	67993: Nudt12 (383) 2.73	94227: Pi15(45) 2.95
151	21826: Thbs2 (1566) 5.15	67092: Gatm (379) -2.5	14824: Grn (592) 2.45	56014: Olfr70(62) -2.94
152	74148: 1300001I01Rik (465) -1.55	74400: 4933405K07Rik (152) 2.22	19791: Rn18s (366) 2.75	20846: Stat1(317) 2.94
153	14451: Gas1 (852) 2.72	21810: Tgfbi (237) -2.21	67963: Npc2 (378) 2.73	17840: Mup1(979) 2.93
154	22639: Zfa (198) 2.22	66406: Sac3d1 (525) -2.24	23918: Impdh2 (2468) -2.13	23959: Nt5e(58) -2.93
155	18023: Nfe2l1 (744) 1.46	16177: Il1r1 (659) 2.38	16739: L1Md-Tf8 (585) 2.45	14238: Foxf2(132) -2.92
156	11475: Acta2 (336) -3.68	20310: Cxcl2 (117) 6.48	12091: Glb1 (5904) -2.12	54598: Calcrl(80) 2.91
157	11492: Adam19 (228) -1.54	76933: Ifi27 (153) 5.38	223254: Farp1 (1257) -2.23	326619: Hist1h4a(77) -2.91
158	13660: Ehd1 (551) -1.61	23792: Adam23 (99) 3.53	20464: Sim1 (12186) 2.14	18301: Fxyd5(269) -2.9
159	12332: Capg (1313) 1.57	107765: Ankrd1 (1757) -2.31	74200: 2810403A07Rik (16619) 2.15	76992: 1700066J24Rik(20) -2.89
160	14980: H2-L (2343) 1.86	70556: 5730438N18Rik (300) -2.23	258430: Olfr938 (126) 4.48	67286: Rabl5(490) 2.88
161	70556: 5730438N18Rik (300) -2.23	11532: Adh5 (821) 2.87	22678: Zfp2 (87) 6.28	78892: Crispld2(118) 2.88
162	50915: Grb14 (541) 1.82	223254: Farp1 (1257) -2.23	16177: Il1r1 (659) 2.38	16625: Serpina3c(40) 2.88

163	26921: Map4k4 (465) 2.5	22639: Zfa (198) 2.22	17775: Laptm4a (2661) 2.1	11532: Adh5(821) 2.87
164	16627: Klra1 (443) -2.65	20944: Svs5 (1528) -2.26	12457: Ccrn4l (373) -2.69	AF192382: AF192382(84) -2.87
165	18301: Fxyd5 (269) -2.9	15228: Foxg1 (400) -2.23	12338: Capn6 (427) 2.58	56533: Rgs17(57) -2.86
166	20271: Scn5a (421) -1.76	18214: Ddr2 (1385) 2.4	11702: Amd1 (592) -2.4	24059: Slco2a1(341) 2.86
167	66181: Nola3 (847) -1.59	21384: Tbx15 (1237) -2.37	67893: Tmem86a (305) 2.83	11830: Aqp5(53) 2.86
168	223254: Farp1 (1257) -2.23	57028: Pdxp (156) -2.33	56504: Stk23 (79) 6.9	15200: Dtr(368) -2.85
169	71791: Cpa4 (171) 1.75	70676: Gulp1 (165) 2.41	20207: Saa-ps (3382) 2.07	11855: Arhgap5(232) 2.85
170	15228: Foxg1 (400) -2.23	12835: Col6a3 (251) -2.28	12890: Cplx2 (435) 2.55	67893: Tmem86a(305) 2.83
171	20607: Sstr3 (362) 2.67	19883: Rora (147) 3.12	20607: Sstr3 (362) 2.67	23828: Bves(103) -2.83
172	26419: Mapk8 (475) 2.81	13400: Dm15 (310) 3.99	26921: Map4k4 (465) 2.5	70673: Prdm16(54) -2.82
173	97541: Qars (707) -1.47	14456: Gas6 (714) 2.63	65100: Zic5 (765) -2.29	26419: Mapk8(475) 2.81
174	18049: Ngfb (337) -2.6	68252: A030007L17Rik (453) 2.78	23794: Adamts5 (1474) 2.14	16773: Lama2(295) -2.81
175	14732: Gpam (317) -1.56	23876: Fbln5 (1186) 2.09	19063: Ppt1 (926) 2.23	11810: Apobec1(60) 2.81
176	14871: Gstt1 (574) 4.16	14573: Gdnf (240) -2.27	18301: Fxyd5 (269) -2.9	94226: Edg8(98) 2.8
177	13025: Ctla2b (173) 2.33	12793: Cnih (1040) -2.07	83433: Trem2b (8219) 2.08	64817: Svep1(738) 2.79
178	20810: Srm (2355) -1.98	67834: Idh3a (771) -2.19	16740: L1Md-Tf9 (452) 2.5	16398: Itga2(47) 2.79
179	66139: 1110002H13Rik (120) -1.72	15208: Hes5 (76) 2.64	14313: Fst (920) 2.23	17858: Mx2(135) 2.79
180	26360: Angptl2 (362) -1.81	21923: Tnc (1616) -2.26	20311: Cxcl5 (11367) 2.08	13004: Cspg3(114) 2.79
181	15211: Hexa (883) 1.38	67938: Mylc2b (1893) 2.19	19225: Ptgs2 (339) -2.69	68252: A030007L17Rik(453) 2.78
182	68114: Mum1 (312) 1.64	15024: H2-T10 (192) 2.17	16773: Lama2 (295) -2.81	14525: Gcet(67) 2.77
183	12476: Cd151 (750) -1.47	19225: Ptgs2 (339) -2.69	14432: Gap43 (225) -3.09	56277: Tmem45a(89) 2.77
184	74359: 4931414P19Rik (309) 2.57	20392: Sgce (359) 3.75	16399: Itga2b (177) 3.42	14962: H2-Bf(61) 2.77
185	56278: Gkap1 (168) -1.73	56338: Txnip (329) 2.51	24110: Usp18 (242) 2.99	17142: Magea6(52) -2.77
186	54123: Irf7 (269) 7.51	54384: Mtmr7 (23) -2.57	12577: Cdkn1c (681) -2.29	73353: Arpm2(668) -2.77
187	22367: Vrk1 (566) 1.45	74558: Gvin1 (291) 3.88	20202: S100a9 (967) -2.2	19791: Rn18s(366) 2.75
188	624217: Gag (1263) 1.6	71900: Tmem106b (398) 2.33	20378: Frzb (237) 3.01	66473: Ctrb1(132) 2.75
189	18035: Nfkbia (1224) 1.65	58235: Pvrl1 (86) -2.56	56316: Ggcx (3797) 2.04	70747: Tspan2(139) -2.74
190	15024: H2-T10 (192) 2.17	56264: Cpxm1 (271) 2.09	12892: Cpo (612) 2.33	21928: Tnfaip2(691) 2.73
191	67775: Rtp4 (190) 5.85	14570: Arhgdig (79) -4.73	13202: Ddt (800) 2.24	67993: Nudt12(383) 2.73
192	74480: Samd4 (363) -1.73	73353: Arpm2 (668) -2.77	21345: Tagln (13068) -2.06	67963: Npc2(378) 2.73
193	20379: Sfrp4 (117) 24.97	67974: 5730405I09Rik (526) -2.15	16997: Ltbp2 (725) 2.26	66175: Mustn1(496) 2.72
194	13874: Ereg (583) -3.19	26367: Ceacam2 (14279) 2.27	27217: Mixl1 (1258) -2.12	20393: Sgk(1758) -2.72
195	66231: Thoc7 (462) -1.66	107449: Unc5b (784) -2.22	22441: Xlr (45) -15.13	14451: Gas1(852) 2.72
196	19944: Rpl29 (10769) -1.77	22771: Zic1 (374) -2.2	107449: Unc5b (784) -2.22	66863: Lztr1(264) 2.71
197	219072: D14Ertd500e (222) -1.99	12424: Cck (478) 2.62	12579: Cdkn2b (731) 2.23	20341: Selenbp1(141) 2.71
198	66113: Apoa5 (3127) -1.54	19025: Ppgb (1617) 2.06	20716: Serpina3n (84) 5.9	110006: Gus(81) -2.71
199	78304: Lsmd1 (1086) -1.62	22240: Dpysl3 (751) -2.39	16733: L1Md-Tf23 (697) 2.24	14009: Etv1(71) 2.71
200	19352: Rabggtb (581) -1.36	68836: Mrpl52 (968) -2	114566: Krt2-20 (678) 2.25	12967: Crygd(42) 2.7
201	70699: Nup205 (249) -1.51	56417: Adar (141) 2.12	19025: Ppgb (1617) 2.06	76477: Pcolce2(84) 2.7
202	207952: Klhl25 (157) 1.75	15505: Hsp105 (827) -2.18	83397: Akap12 (876) -2.17	18124: Nr4a3(47) 2.69
203	19711: Resp18 (470) -1.45	12578: Cdkn2a (248) 2.14	15505: Hsp105 (827) -2.18	19401: Rara(279) 2.69
204	56417: Adar (141) 2.12	17131: Madh7 (500) -2.17	18049: Ngfb (337) -2.6	18811: Plf(1863) -2.69
205	21928: Tnfaip2 (691) 2.73	12892: Cpo (612) 2.33	15006: H2-Q1 (573) 2.29	19225: Ptgs2(339) -2.69

206	71941: 2310051N18Rik (12991) 1.89	18854: Pml (239) 2.22	67834: Idh3a (771) -2.19	12457: Ccrn4l(373) -2.69
207	67834: Idh3a (771) -2.19	11855: Arhgap5 (232) 2.85	67092: Gatm (379) -2.5	66447: Mgst3(280) 2.68
208	20944: Svs5 (1528) -2.26	83397: Akap12 (876) -2.17	19173: Psmb5 (1755) -2.04	20607: Sstr3(362) 2.67
209	99650: 4933434E20Rik (727) -1.43	15227: Foxf1a (183) -3.79	211401: Mtss1 (6333) -1.99	21893: Tlm(2271) 2.67
210	72962: Ecgf1 (307) -1.66	16323: Inhba (454) -2.01	23876: Fbln5 (1186) 2.09	27056: Irf5(101) -2.67
211	18674: Slc25a3 (5083) -1.44	27424: Klra16 (80) -2.58	76743: D230044M03Rik (5575) 1.98	16627: Klra1(443) -2.65
212	16399: Itga2b (177) 3.42	15039: H2-T22 (1535) 1.99	11845: Arf6 (1267) 2.07	27423: Klra15(86) -2.65
213	12842: Col1a1 (32794) 1.71	21825: Thbs1 (4229) -2.14	19401: Rara (279) 2.69	22271: Upp1(101) 2.65
214	67974: 5730405I09Rik (526) -2.15	14313: Fst (920) 2.23	66447: Mgst3 (280) 2.68	16630: Klra12(187) -2.64
215	54196: Pabpn1 (1818) -1.46	27217: Mixl1 (1258) -2.12	66054: Cndp2 (437) -2.38	15208: Hes5(76) 2.64
216	11845: Arf6 (1267) 2.07	56760: Clec1b (120) 3.26	20810: Srm (2355) -1.98	14456: Gas6(714) 2.63
217	67938: Mylc2b (1893) 2.19	20311: Cxcl5 (11367) 2.08	11855: Arhgap5 (232) 2.85	12424: Cck(478) 2.62
218	56795: Arm1 (156) -1.3	110006: Gus (81) -2.71	20308: Ccl9 (203) 3	19373: Rag1(86) 2.62
219	69034: 1810010E01Rik (1645) -1.65	17775: Laptm4a (2661) 2.1	66058: 0610011I04Rik (4094) 1.96	18111: Nnat(109) 2.61
220	20311: Cxcl5 (11367) 2.08	NM_014195: L1Md-Tf29 (505) 3.15	15439: Hp (2965) 1.95	18426: Ovol1(42) 2.61
221	56200: Ddx21 (455) -1.67	17285: Meox1 (43) 2.32	66863: Lztr1 (264) 2.71	18049: Ngfb(337) -2.6
222	22791: Dnajc2 (832) -1.68	258604: Olfr970 (368) -2.03	74359: 4931414P19Rik (309) 2.57	14118: Fbn1(908) 2.59
223	19173: Psmb5 (1755) -2.04	11845: Arf6 (1267) 2.07	12793: Cnih (1040) -2.07	12338: Capn6(427) 2.58
224	18727: Pira4 (460) 3.16	14205: Figf (568) 2.23	14205: Figf (568) 2.23	15412: Hoxb4(117) -2.58
225	13198: Ddit3 (455) 1.89	67149: 2610200G18Rik (316) -2.21	20377: Sfrp1 (1516) 2.01	27424: Klra16(80) -2.58
226	56418: Ykt6 (881) -2.38	18153: Npn2 (196) 2.36	56338: Txnip (329) 2.51	74359: 4931414P19Rik(309) 2.57
227	12606: Cebpa (3460) 1.53	72654: Ccdc12 (668) -1.99	19174: Psmb5-ps (1463) -2	54384: Mtmr7(23) -2.57
228	94275: Maged1 (2993) 1.82	70110: Ifi35 (377) 2.28	70025: Bach (864) -2.1	16737: L1Md-Tf5(1009) 2.56
229	107765: Ankrd1 (1757) -2.31	12338: Capn6 (427) 2.58	66480: Rpl15 (18626) -1.97	16145: Igtp(168) 2.56
230	80861: D11Lgp2e (109) 8.02	21788: Tfpi (82) -3.72	66406: Sac3d1 (525) -2.24	16738: L1Md-Tf6(548) 2.56
231	12578: Cdkn2a (248) 2.14	16204: Fabp6 (392) -2.09	15039: H2-T22 (1535) 1.99	58235: Pvrl1(86) -2.56
232	66447: Mgst3 (280) 2.68	15439: Hp (2965) 1.95	16582: Kifc3 (544) -2.21	260409: Cdc42ep3(503) 2.56
233	20716: Serpina3n (84) 5.9	73287: 1700040L02Rik (475) -2.14	192216: Tm4sf10 (69) -6.61	22644: Rnf103(4139) -2.55
234	66248: Alg5 (840) -1.49	97122: Hist2h4 (1607) 2.26	18365: Olfr64 (7100) -1.93	12890: Cplx2(435) 2.55
235	53600: Timm23 (1505) -1.93	16739: L1Md-Tf8 (585) 2.45	66695: Aspn (155) 3.29	19266: Ptprd(91) -2.55
236	14958: H1f0 (649) 1.57	58185: Rsad2 (103) 3.38	53622: Krt2-18 (62) -7.61	56532: Ripk3(215) -2.55
237	22024: Crisp2 (93) -1.49	19173: Psmb5 (1755) -2.04	66340: Psenen (14897) 1.94	286940: Flnb(891) -2.55
238	22635: Zan (218) 1.37	97122: Hist2h4 (1102) 2.37	12978: Csf1r (1403) 1.99	15229: Foxd1(151) -2.55
239	69536: Hemk1 (243) 1.35	12269: C4bp (56) 3.89	71584: Gdpd2 (174) 3.07	15040: H2-T23(142) 2.54
240	13806: Eno1 (14056) -1.75	58802: Kcnmb4 (126) 2.06	14824: Grn (1107) 2.02	78901: 4833444C15Rik(82) 2.53
241	69546: Mapk1ip1 (464) -1.78	73340: Nptxr (742) 2.04	71900: Tmem106b (398) 2.33	20402: Sh3bp3(179) -2.53
242	94092: Trim16 (918) 1.31	54526: Syt10 (112) -3.68	11933: Atp1b3 (425) -2.32	171095: Il17rc(123) -2.53
243	56297: Arl6 (439) -1.32	23962: Oasl2 (279) 3.94	16644: Kng (1226) 1.99	17035: Lxn(2296) 2.53
244	69590: 2310016C16Rik (1876) 1.48	110253: Triobp (1191) 1.91	14362: Fzd1 (383) 2.34	11717: Ampd3(109) 2.53

245	60409: Trappc4 (1190) -1.67	70025: Bach (864) -2.1	74987: 4930468A15Rik (1225) 1.98	19735: Rgs2(108) 2.53
246	18150: Npm3 (364) -1.71	22441: Xlr (45) -15.13	78600: Pde6h (61) 7.34	16734: L1Md-Tf26(1023) 2.51
247	20765: Sprr2k (109) 3.4	76856: Catsper3 (63) 2.47	15982: Ifrd1 (1052) 2.01	78267: Klhdc8b(944) 2.51
248	56529: Sec11l1 (1267) 1.27	16730: L1Md-Tf14 (302) 2.42	12630: Cfi (2646) 1.89	56338: Txnip(329) 2.51
249	30962: Slc7a9 (85) 1.88	13591: Ebf1 (269) 1.95	Z80833: Z80833 (1959) -1.92	20690: Spam(63) -2.51
250	59042: Cope (1766) 1.44	219072: D14Ertd500e (222) -1.99	18616: Peg3 (141) -3.35	16732: L1Md-Tf18(701) 2.51
251	80280: Cdk5rap3 (586) 1.62	67834: Idh3a (1504) -1.94	16731: L1Md-Tf17 (758) 2.06	26921: Map4k4(465) 2.5
252	93692: Glrx1 (812) -1.82	20810: Srm (2355) -1.98	67834: Idh3a (1504) -1.94	16740: L1Md-Tf9(452) 2.5
253	17279: Melk (1024) -1.64	54613: Siat10 (504) 1.95	99899: Ifi44 (115) 3.79	67092: Gatm(379) -2.5
254	17775: Laptm4a (2661) 2.1	78092: 4921511M17Rik (81) 2.03	15039: H2-T22 (1419) 1.94	66996: Ceacam11(172) 2.49
255	12306: Anxa2 (5837) -1.45	11998: Avp (135) 1.91	68836: Mrpl52 (968) -2	63913: Niban(136) 2.49
256	12835: Col6a3 (251) -2.28	60363: Cldn15 (56) 2.32	53600: Timm23 (1505) -1.93	78795: Armc9(24) 2.48
257	68607: Serhl (803) -1.82	16019: Igh-6 (1713) -2.23	71941: 2310051N18Rik (12991) 1.89	22418: Wnt5a(105) -2.48
258	26442: Psma5 (1784) -1.5	14824: Grn (1107) 2.02	73340: Nptxr (742) 2.04	26903: Dysf(45) -2.47
259	17133: Maff (287) -1.97	65100: Zic5 (765) -2.29	67974: 5730405I09Rik (526) -2.15	12296: Cacnb2(46) 2.47
260	15039: H2-T22 (1419) 1.94	19174: Psmb5-ps (1463) -2	17131: Madh7 (500) -2.17	214682: Myo3a(88) 2.47
261	50759: Fbxo16 (103) -1.91	22696: Zfp37 (180) 2.15	16730: L1Md-Tf14 (302) 2.42	76856: Catsper3(63) 2.47
262	19174: Psmb5-ps (1463) -2	73068: Fut11 (137) -2.01	19213: Ptf1a (1419) 1.92	66410: Mterfd1(157) 2.46
263	22302: V2r11 (182) -1.59	12409: Cbr2 (326) 2.01	22302: V2r11 (428) -2.22	16736: L1Md-Tf30(1021) 2.46
264	260409: Cdc42ep3 (503) 2.56	20308: Ccl9 (203) 3	15039: H2-T22 (1050) 1.97	20364: Sepw1(1861) 2.46
265	56772: Mllt11 (970) -1.71	12091: Glb1 (5904) -2.12	70110: Ifi35 (377) 2.28	13807: Eno2(87) 2.45
266	67843: Slc35a4 (707) -1.69	16582: Kifc3 (544) -2.21	27081: Zfp275 (131) 3.38	14824: Grn(592) 2.45
267	22634: Plagl1 (201) 4.12	19087: Prkar2a (356) -2.24	16008: Igfbp2 (384) -2.26	16739: L1Md-Tf8(585) 2.45
268	71227: 4933429D11Rik (98) 1.59	15982: Ifrd1 (1052) 2.01	14200: Fhl2 (844) 1.99	11576: Afp(34) -2.45
269	22632: Yy1 (951) 1.58	67138: Herc5 (89) 2.29	11431: Acp1 (907) 1.98	68265: Iqcf3(966) 2.45
270	73340: Nptxr (742) 2.04	68709: Cilp2 (82) 2.39	17523: Mpo (905) 1.98	73582: 1700106N22Rik(128) 2.44
271	26905: Eif2s3x (1008) -1.41	11703: Amd2 (730) -1.98	13423: Dnase2a (2074) 1.87	12494: Cd38(79) 2.43
272	13423: Dnase2a (2074) 1.87	68038: 3110023E09Rik (116) -2.16	11461: Actb (36433) -1.89	52668: D12Ertd647e(1749) 2.42
273	14118: Fbn1 (908) 2.59	15115: Hars (850) -1.9	16425: Itih2 (456) 2.17	16819: Lcn2(1153) 2.42
274	14356: Fxc1 (548) -1.39	16145: Igtp (168) 2.56	14980: H2-L (2343) 1.86	16730: L1Md-Tf14(302) 2.42
275	69035: Zdhhc3 (845) -1.65	11431: Acp1 (907) 1.98	18655: Pgk1 (1657) -1.89	70676: Gulp1(165) 2.41
276	73068: Fut11 (137) -2.01	56277: Tmem45a (89) 2.77	15019: H2-Q8 (755) 2.01	22228: Ucp2(70) 2.41
277	14573: Gdnf (240) -2.27	17133: Maff (287) -1.97	15228: Foxg1 (400) -2.23	236539: Phgdh(102) -2.41
278	16475: Jub (306) -1.6	16331: Inpp5d (211) 2.01	19883: Rora (147) 3.12	217588: Mbip(174) 2.41
279	94192: C1galt1 (323) 1.69	13807: Eno2 (87) 2.45	54526: Syt10 (112) -3.68	77994: D730043B02Rik(64) -2.4
280	19242: Ptn (506) 3.18	AF037046: AF037046 (219) 2.24	19659: Rbp1 (1095) 1.94	54396: Iigp2(251) 2.4
281	54216: Pcdh7 (520) 1.88	112407: Egln3 (152) 5.03	70859: 4921509B22Rik (622) 2.05	74894: 4930442H23Rik(58) 2.4
282	67895: Ppa1 (1028) -1.66	15019: H2-Q8 (755) 2.01	21825: Thbs1 (6216) -1.84	18214: Ddr2(1385) 2.4
283	11703: Amd2 (730) -1.98	17071: Ly6f (430) 1.98	50772: Mapk6 (1288) -1.91	11702: Amd1(592) -2.4

284	16177: Il1r1 (659) 2.38	27055: Fkbp9 (275) -2.15	12505: Cd44 (2353) -1.85	22240: Dpysl3(751) -2.39
285	27423: Klra15 (86) -2.65	18812: Plf2 (649) -5.41	73287: 1700040L02Rik (475) -2.14	68709: Cilp2(82) 2.39
286	11431: Acp1 (907) 1.98	22644: Rnf103 (4139) -2.55	67738: Ppid (2054) -1.86	AF030001: AF030001(71) 2.39
287	17131: Madh7 (500) -2.17	217069: Trim25 (223) 2.19	76799: 2510006D16Rik (24202) 1.86	71690: Esm1(75) -2.38
288	56405: Dusp14 (501) -1.41	15039: H2-T22 (1419) 1.94	56213: Prss11 (1931) -1.86	66054: Cndp2(437) -2.38
289	103551: E130012A19Rik (560) 1.42	53600: Timm23 (1505) -1.93	15269: Hist4 (4499) 1.83	54473: Tollip(57) -2.38
290	67952: Tomm20 (2655) -1.71	78795: Armc9 (24) 2.48	114615: Elac1 (2853) -1.82	56418: Ykt6(881) -2.38
291	27217: Mixl1 (1258) -2.12	56199: Abcb10 (340) -2.04	110253: Triobp (1191) 1.91	19054: Ppp2r3a(37) -2.38
292	23892: Cktsf1b1 (344) 4.82	22678: Zfp2 (87) 6.28	94275: Maged1 (2993) 1.82	66573: Dzip1(74) 2.38
293	14824: Grn (1107) 2.02	16732: L1Md-Tf18 (701) 2.51	11703: Amd2 (730) -1.98	16177: Il1r1(659) 2.38
294	78092: 4921511M17Rik (81) 2.03	15039: H2-T22 (1050) 1.97	16065: Igh-VS107 (5824) 1.82	97122: Hist2h4(1102) 2.37
295	21923: Tnc (1616) -2.26	54352: Irx5 (108) -3.1	21417: Zfhx1a (322) -2.3	21384: Tbx15(1237) -2.37
296	27058: Srp9 (1038) 1.52	22004: Tpm2 (418) -1.89	19087: Prkar2a (356) -2.24	13169: Dbnl(117) -2.36
297	14208: Ppm1g (129) -1.81	66480: Rpl15 (18626) -1.97	15051: H2-T9 (1141) 1.9	18549: Pcsk2(42) 2.36
298	57028: Pdxp (156) -2.33	18655: Pgk1 (1657) -1.89	75768: 4833422M21Rik (3217) 1.81	18153: Npn2(196) 2.36
299	12409: Cbr2 (326) 2.01	16736: L1Md-Tf30 (1021) 2.46	76291: 1110003O08Rik (10234) 1.83	26358: Aldh1a7(38) 2.35
300	77590: 4631426J05Rik (4390) 1.22	16738: L1Md-Tf6 (548) 2.56	20090: Rps29 (11930) 1.83	13885: Es10(1973) -2.35

7) List of significant Gene Ontology categories for Nickel time course

Table S3: Gene Ontology categories that had a *p*-value<0.005 for each of the 4 tested methods, listing category (# significant genes in category), and *p*-value.

Time	FOLD	IBMT	SMT	T-test
03 hr	oxidoreductase activity\, acting on the CH-NH group of donors\, NAD or NADP as acceptor (2) 2.18E-03	G-protein coupled receptor kinase activity (2) 1.23E-03	G-protein coupled receptor kinase activity (2) 1.19E-03	oxidoreductase activity\, acting on the CH-NH group of donors\, NAD or NADP as acceptor (2) 2.35E-03
03 hr	peptide receptor activity (4) 4.4E-03	oxidoreductase activity\, acting on the CH-NH group of donors\, NAD or NADP as acceptor (2) 2.44E-03	oxidoreductase activity\, acting on the CH-NH group of donors\, NAD or NADP as acceptor (2) 2.35E-03	protein-lysine 6-oxidase activity (2) 2.35E-03
03 hr	peptide receptor activity\, G-protein coupled (4) 4.4E-03	protein-lysine 6-oxidase activity (2) 2.44E-03	segment specification (2) 4.01E-03	
03 hr		segment specification (2) 4.16E-03		
08 hr	immune response (16) 7E-05	extracellular (46) 3.53E-05	glycolysis (5) 2.11E-04	glucose metabolism (5) 1.97E-03
08 hr	extracellular space (39) 1.69E-04	extracellular space (41) 7.62E-05	glucose metabolism (6) 3.35E-04	cartilage condensation (2) 2.72E-03
08 hr	extracellular (42) 3.35E-04	glycolysis (5) 2.37E-04	regulation of angiogenesis (3) 3.8E-04	hearing (3) 3.04E-03
08 hr	defense response (17) 3.78E-04	oxidoreductase activity\, acting on paired donors\, with incorporation or reduction of molecular oxygen (6) 3.11E-04	glucose catabolism (5) 5.15E-04	blood coagulation (4) 3.28E-03
08 hr	regulation of angiogenesis (3) 4E-04	glucose metabolism (6) 3.83E-04	alcohol catabolism (5) 6.03E-04	hemostasis (4) 3.72E-03
08 hr	response to biotic stimulus (17) 1.55E-03	regulation of angiogenesis (3) 4.09E-04	hexose catabolism (5) 6.03E-04	perception of sound (3) 3.74E-03
08 hr	thyroid hormone receptor activity (2) 1.57E-03	glucose catabolism (5) 5.78E-04	monosaccharide catabolism (5) 6.03E-04	

08 hr	transcriptional repressor activity (4) 1.71E-03	oxidoreductase activity\, acting on paired donors\, with incorporation or reduction of molecular oxygen\, 2-oxoglutarate as one donor\, and incorporation of one atom each of oxygen into both donors (3) 6.66E-04	oxidoreductase activity\, acting on paired donors\, with incorporation or reduction of molecular oxygen\, 2-oxoglutarate as one donor\, and incorporation of one atom each of oxygen into both donors (3) 6.21E-04	
08 hr	lymphocyte proliferation (3) 1.76E-03	alcohol catabolism (5) 6.76E-04	main pathways of carbohydrate metabolism (6) 6.25E-04	
08 hr	glycolysis (4) 2.4E-03	hexose catabolism (5) 6.76E-04	carbohydrate catabolism (5) 1.07E-03	
08 hr	intermediate filament (5) 2.53E-03	monosaccharide catabolism (5) 6.76E-04	hexose metabolism (6) 1.32E-03	
08 hr	intermediate filament cytoskeleton (5) 2.53E-03	main pathways of carbohydrate metabolism (6) 7.13E-04	monosaccharide metabolism (6) 1.45E-03	
08 hr	response to wounding (7) 2.57E-03	carbohydrate catabolism (5) 1.2E-03	extracellular (40) 1.66E-03	
08 hr	glucose metabolism (5) 2.66E-03	hexose metabolism (6) 1.5E-03	oxidoreductase activity\, acting on single donors with incorporation of molecular oxygen\, incorporation of two atoms of oxygen (3) 1.74E-03	
08 hr	nucleotide receptor activity (3) 2.91E-03	monosaccharide metabolism (6) 1.65E-03	extracellular space (36) 1.87E-03	
08 hr	nucleotide receptor activity\, G-protein coupled (3) 2.91E-03	oxidoreductase activity\, acting on single donors with incorporation of molecular oxygen\, incorporation of two atoms of oxygen (3) 1.86E-03	oxidoreductase activity\, acting on paired donors\, with incorporation or reduction of molecular oxygen (5) 2.1E-03	
08 hr	protein domain specific binding (3) 2.91E-03	oxidoreductase activity\, acting on single donors with incorporation of molecular oxygen (3) 2.44E-03	oxidoreductase activity\, acting on single donors with incorporation of molecular oxygen (3) 2.28E-03	
08 hr	purinergic nucleotide receptor activity (3) 2.91E-03	blood coagulation (4) 4.32E-03	blood coagulation (4) 3.95E-03	
08 hr	purinergic nucleotide receptor activity\, G-protein coupled (3) 2.91E-03	energy derivation by oxidation of organic compounds (6) 4.88E-03	energy derivation by oxidation of organic compounds (6) 4.32E-03	
08 hr	myeloid blood cell differentiation (3) 3.69E-03	hemostasis (4) 4.9E-03	hemostasis (4) 4.48E-03	
08 hr	main pathways of carbohydrate metabolism (5) 4.38E-03		receptor binding (11) 4.78E-03	
08 hr	negative regulation of transcription (5) 4.38E-03			
08 hr	T-cell activation (3) 4.53E-03			
08 hr	glucose catabolism (4) 4.75E-03			
08 hr	lymphocyte activation (4) 4.75E-03			
08 hr	receptor binding (11) 4.78E-03			
08 hr	chemotaxis (5) 4.79E-03			
08 hr	taxis (5) 4.79E-03			
24 hr	carbohydrate catabolism (9) 8.75E-09	carbohydrate catabolism (10) 1.38E-09	carbohydrate catabolism (10) 1.51E-09	carbohydrate catabolism (9) 3.96E-08
24 hr	glycolysis (7) 2.28E-07	glucose catabolism (8) 1.22E-07	glucose catabolism (8) 1.31E-07	glucose metabolism (8) 2.79E-06
24 hr	glucose metabolism (8) 7.57E-07	glucose metabolism (9) 1.49E-07	glucose metabolism (9) 1.62E-07	glucose catabolism (7) 2.84E-06
24 hr	glucose catabolism (7) 8.95E-07	alcohol catabolism (8) 1.61E-07	alcohol catabolism (8) 1.73E-07	alcohol catabolism (7) 3.61E-06
24 hr	alcohol catabolism (7) 1.14E-06	hexose catabolism (8) 1.61E-07	hexose catabolism (8) 1.73E-07	hexose catabolism (7) 3.61E-06
24 hr	hexose catabolism (7) 1.14E-06	monosaccharide catabolism (8) 1.61E-07	monosaccharide catabolism (8) 1.73E-07	monosaccharide catabolism (7) 3.61E-06

24 hr	monosaccharide catabolism (7) 1.14E-06	main pathways of carbohydrate metabolism (9) 4.18E-07	main pathways of carbohydrate metabolism (9) 4.52E-07	hexose metabolism (8) 1.97E-05
24 hr	main pathways of carbohydrate metabolism (8) 1.87E-06	hexose metabolism (9) 1.44E-06	hexose metabolism (9) 1.56E-06	monosaccharide metabolism (8) 2.26E-05
24 hr	electrochemical potential-driven transporter activity (9) 3.91E-06	monosaccharide metabolism (9) 1.7E-06	monosaccharide metabolism (9) 1.83E-06	extracellular matrix (12) 2.63E-05
24 hr	porter activity (9) 3.91E-06	glycolysis (6) 9.87E-06	glycolysis (6) 1.04E-05	main pathways of carbohydrate metabolism (7) 6.44E-05
24 hr	hexose metabolism (8) 5.55E-06	energy derivation by oxidation of organic compounds (9) 1.05E-05	energy derivation by oxidation of organic compounds (9) 1.13E-05	carbohydrate metabolism (13) 9.67E-05
24 hr	monosaccharide metabolism (8) 6.4E-06	carbohydrate metabolism (14) 1.33E-05	carbohydrate metabolism (14) 1.48E-05	glycolysis (5) 1.79E-04
24 hr	carbohydrate metabolism (13) 1.55E-05	alcohol metabolism (10) 3.45E-05	alcohol metabolism (10) 3.74E-05	energy pathways (8) 3.4E-04
24 hr	energy derivation by oxidation of organic compounds (8) 3.18E-05	energy pathways (9) 3.98E-05	energy pathways (9) 4.28E-05	energy derivation by oxidation of organic compounds (7) 6.78E-04
24 hr	alcohol metabolism (9) 7.41E-05	extracellular matrix (11) 7.31E-05	extracellular matrix (11) 8.63E-05	extracellular (41) 6.94E-04
24 hr	energy pathways (8) 1.04E-04	catabolism (19) 2.58E-04	intermediate filament (6) 2.73E-04	alcohol metabolism (8) 1.33E-03
24 hr	endopeptidase inhibitor activity (7) 1.71E-04	glutamate-cysteine ligase activity (2) 4.4E-04	intermediate filament cytoskeleton (6) 2.73E-04	coenzyme metabolism (6) 2.28E-03
24 hr	protease inhibitor activity (7) 1.71E-04	enzyme inhibitor activity (9) 6.89E-04	glutamate-cysteine ligase activity (2) 4.56E-04	iron ion binding (3) 2.59E-03
24 hr	enzyme inhibitor activity (9) 2.57E-04	extracellular (39) 7.16E-04	extracellular (40) 5.2E-04	extracellular space (35) 3.1E-03
24 hr	centriole (2) 1.01E-03	centriole (2) 1.3E-03	enzyme inhibitor activity (9) 7.82E-04	catabolism (17) 3.17E-03
24 hr	nitric oxide mediated signal transduction (2) 1.02E-03	glutathione biosynthesis (2) 1.31E-03	catabolism (18) 8.18E-04	structural constituent of cytoskeleton (6) 3.48E-03
24 hr	intermediate filament (5) 1.1E-03	substrate-bound cell migration (2) 1.31E-03	glutathione biosynthesis (2) 1.33E-03	blood coagulation (4) 3.49E-03
24 hr	intermediate filament cytoskeleton (5) 1.1E-03	intermediate filament (5) 1.93E-03	substrate-bound cell migration (2) 1.33E-03	hemostasis (4) 3.96E-03
24 hr	serine-type endopeptidase inhibitor activity (5) 1.46E-03	intermediate filament cytoskeleton (5) 1.93E-03	centriole (2) 1.35E-03	NADPH regeneration (2) 4.62E-03
24 hr	iron ion binding (3) 1.53E-03	extracellular space (34) 1.94E-03	extracellular space (35) 1.36E-03	pentose-phosphate shunt (2) 4.62E-03
24 hr	defense/immunity protein activity (6) 2.45E-03	endopeptidase inhibitor activity (6) 2.3E-03	iron ion binding (3) 2.35E-03	
24 hr	extracellular matrix (8) 2.56E-03	protease inhibitor activity (6) 2.3E-03	endopeptidase inhibitor activity (6) 2.52E-03	
24 hr	carrier activity (11) 2.78E-03	aldolase activity (2) 2.57E-03	protease inhibitor activity (6) 2.52E-03	
24 hr	digestion (2) 3.31E-03	structural constituent of cytoskeleton (6) 2.69E-03	aldolase activity (2) 2.66E-03	
24 hr	positive regulation of lymphocyte proliferation (2) 3.31E-03	electrochemical potential-driven transporter activity (6) 3.12E-03	structural constituent of cytoskeleton (6) 2.94E-03	
24 hr	enzyme regulator activity (12) 3.36E-03	porter activity (6) 3.12E-03	electrochemical potential-driven transporter activity (6) 3.41E-03	
24 hr	extracellular (33) 3.84E-03	NADPH regeneration (2) 4.24E-03	porter activity (6) 3.41E-03	
24 hr	neurotransmitter transporter activity (2) 4.82E-03	pentose-phosphate shunt (2) 4.24E-03	NADPH regeneration (2) 4.31E-03	
24 hr	neurotransmitter\:sodium symporter activity (2) 4.82E-03	positive regulation of lymphocyte proliferation (2) 4.24E-03	pentose-phosphate shunt (2) 4.31E-03	
24 hr		defense/immunity protein activity (6) 4.73E-03	positive regulation of lymphocyte proliferation (2) 4.31E-03	

48 hr	intermediate filament (6) 1.54E-04	extracellular (45) 1.07E-05	extracellular (41) 7.55E-05	response to fungi (2) 4.66E-04
48 hr	intermediate filament cytoskeleton (6) 1.54E-04	extracellular space (41) 1.21E-05	extracellular space (37) 1.05E-04	response to stress (15) 1.22E-03
48 hr	antiporter activity (3) 7.92E-04	hematopoietin/interferon-class (D200-domain) cytokine receptor activity (5) 4.3E-04	response to fungi (2) 4.34E-04	tRNA methyltransferase activity (2) 1.37E-03
48 hr	glucose metabolism (5) 1.04E-03	response to fungi (2) 4.92E-04	IgE binding (2) 4.4E-04	endopeptidase inhibitor activity (6) 2.63E-03
48 hr	glycolysis (4) 1.1E-03	ribonucleoprotein binding (2) 5.13E-04	extracellular matrix (9) 1.21E-03	protease inhibitor activity (6) 2.63E-03
48 hr	solute\:cation antiporter activity (2) 1.12E-03	carbohydrate catabolism (5) 9.55E-04	embryonic eye morphogenesis (2) 1.28E-03	extracellular space (34) 2.71E-03
48 hr	solute\:hydrogen antiporter activity (2) 1.12E-03	embryonic eye morphogenesis (2) 1.45E-03	eye morphogenesis (sensu Mammalia) (2) 1.28E-03	extracellular (37) 4.02E-03
48 hr	solute\:solute antiporter activity (2) 1.12E-03	eye morphogenesis (sensu Mammalia) (2) 1.45E-03	eye morphogenesis (sensu Vertebrata) (2) 1.28E-03	macrophage activation (2) 4.47E-03
48 hr	main pathways of carbohydrate metabolism (5) 1.74E-03	eye morphogenesis (sensu Vertebrata) (2) 1.45E-03	immunoglobulin binding (2) 2.57E-03	
48 hr	cation homeostasis (4) 1.95E-03	extracellular matrix (9) 1.78E-03	hematopoietin/interferon-class (D200-domain) cytokine receptor activity (4) 2.96E-03	
48 hr	transition metal ion homeostasis (3) 2.02E-03	glycolysis (4) 2.06E-03	oxidoreductase activity\, acting on paired donors\, with incorporation or reduction of molecular oxygen\, reduced flavin or flavoprotein as one donor\, and incorporation of one atom of oxygen (3) 3.45E-03	
48 hr	glucose catabolism (4) 2.22E-03	glucose metabolism (5) 2.21E-03	eye morphogenesis (2) 4.16E-03	
48 hr	lysozyme activity (2) 2.22E-03	interleukin binding (3) 3.48E-03	macrophage activation (2) 4.16E-03	
48 hr	response to pest/pathogen/parasite (8) 2.38E-03	interleukin receptor activity (3) 3.48E-03	response to pest/pathogen/parasite (8) 4.61E-03	
48 hr	alcohol catabolism (4) 2.51E-03	cation homeostasis (4) 3.6E-03	embryonic morphogenesis (3) 4.89E-03	
48 hr	cell ion homeostasis (4) 2.51E-03	main pathways of carbohydrate metabolism (5) 3.65E-03		
48 hr	hexose catabolism (4) 2.51E-03	glucose catabolism (4) 4.09E-03		
48 hr	ion homeostasis (4) 2.51E-03	oxidoreductase activity\, acting on paired donors\, with incorporation or reduction of molecular oxygen\, reduced flavin or flavoprotein as one donor\, and incorporation of one atom of oxygen (3) 4.28E-03		
48 hr	monosaccharide catabolism (4) 2.51E-03	alcohol catabolism (4) 4.61E-03		
48 hr	oxidoreductase activity\, acting on paired donors\, with incorporation or reduction of molecular oxygen\, reduced flavin or flavoprotein as one donor\, and incorporation of one atom of oxygen (3) 2.8E-03	cell ion homeostasis (4) 4.61E-03		
48 hr	hexose metabolism (5) 3.24E-03	hexose catabolism (4) 4.61E-03		
48 hr	response to hypoxia (2) 3.38E-03	ion homeostasis (4) 4.61E-03		
48 hr	monosaccharide metabolism (5) 3.51E-03	monosaccharide catabolism (4) 4.61E-03		
48 hr	carbohydrate catabolism (4) 3.94E-03	eye morphogenesis (2) 4.7E-03		
48 hr	response to wounding (6) 3.95E-03	macrophage activation (2) 4.7E-03		

48 hr	cell adhesion receptor activity (3) 4.06E-03			
48 hr	cell wall catabolism (2) 5E-03			
48 hr	fertilization (2) 5E-03			
72 hr	extracellular (42) 7.89E-06	extracellular space (48) 2.02E-08	extracellular space (49) 6.32E-09	extracellular (44) 1.4E-04
72 hr	oxidoreductase activity\, acting on paired donors\, with incorporation or reduction of molecular oxygen\, reduced flavin or flavoprotein as one donor\, and incorporation of one atom of oxygen (5) 1.1E-05	extracellular (52) 2.34E-08	extracellular (53) 7.55E-09	extracellular space (40) 1.4E-04
72 hr	angiogenesis (7) 1.36E-05	oxidoreductase activity\, acting on paired donors\, with incorporation or reduction of molecular oxygen\, reduced flavin or flavoprotein as one donor\, and incorporation of one atom of oxygen (5) 1.4E-05	oxidoreductase activity\, acting on paired donors\, with incorporation or reduction of molecular oxygen\, reduced flavin or flavoprotein as one donor\, and incorporation of one atom of oxygen (5) 1.46E-05	extracellular matrix (10) 6.95E-04
72 hr	monooxygenase activity (7) 1.91E-05	extracellular matrix (12) 2.41E-05	extracellular matrix (12) 2.41E-05	glutathione metabolism (3) 1.33E-03
72 hr	oxidoreductase activity\, acting on paired donors\, with incorporation or reduction of molecular oxygen (7) 1.91E-05	oxidoreductase activity\, acting on paired donors\, with incorporation or reduction of molecular oxygen (7) 2.65E-05	oxidoreductase activity\, acting on paired donors\, with incorporation or reduction of molecular oxygen (7) 2.8E-05	response to chemical substance (7) 2.33E-03
72 hr	extracellular space (37) 3.22E-05	endopeptidase inhibitor activity (8) 9.72E-05	endopeptidase inhibitor activity (8) 1.03E-04	endopeptidase inhibitor activity (6) 4.46E-03
72 hr	blood vessel development (7) 3.62E-05	protease inhibitor activity (8) 9.72E-05	protease inhibitor activity (8) 1.03E-04	protease inhibitor activity (6) 4.46E-03
72 hr	morphogenesis (22) 7.45E-05	monooxygenase activity (6) 2.5E-04	angiogenesis (6) 2.2E-04	
72 hr	organogenesis (21) 7.47E-05	enzyme inhibitor activity (10) 2.73E-04	monooxygenase activity (6) 2.61E-04	
72 hr	enzyme inhibitor activity (10) 1.8E-04	serine-type endopeptidase inhibitor activity (6) 5.45E-04	enzyme inhibitor activity (10) 2.92E-04	
72 hr	regulation of lymphocyte proliferation (3) 3.01E-04	structural constituent of cytoskeleton (7) 7.44E-04	blood vessel development (6) 4.92E-04	
72 hr	regulation of B-cell proliferation (2) 4.42E-04	glutathione metabolism (3) 1.21E-03	serine-type endopeptidase inhibitor activity (6) 5.69E-04	
72 hr	endopeptidase inhibitor activity (7) 4.56E-04	microsome (6) 1.26E-03	glutathione metabolism (3) 1.24E-03	
72 hr	protease inhibitor activity (7) 4.56E-04	vesicular fraction (6) 1.39E-03	microsome (6) 1.26E-03	
72 hr	cytolysis (3) 4.75E-04	substrate-bound cell migration (2) 1.5E-03	vesicular fraction (6) 1.39E-03	
72 hr	microsome (6) 6.42E-04	plasminogen activator activity (2) 1.52E-03	substrate-bound cell migration (2) 1.53E-03	
72 hr	vesicular fraction (6) 7.08E-04	muscle contraction (5) 3.93E-03	plasminogen activator activity (2) 1.54E-03	
72 hr	muscle development (6) 1.28E-03		morphogenesis (19) 3.87E-03	
72 hr	substrate-bound cell migration (2) 1.31E-03		organogenesis (18) 3.99E-03	
72 hr	lymphocyte proliferation (3) 1.34E-03		structural constituent of cytoskeleton (6) 4.1E-03	
72 hr	plasminogen activator activity (2) 1.37E-03			
72 hr	intermediate filament (5) 1.38E-03			
72 hr	intermediate filament cytoskeleton (5) 1.38E-03			
72 hr	myofibril (4) 2.24E-03			
72 hr	sarcomere (4) 2.24E-03			

72 hr	membrane fraction (8) 2.48E-03			
72 hr	B-cell proliferation (2) 2.58E-03			
72 hr	negative regulation of CDK activity (2) 2.58E-03			
72 hr	muscle contraction (5) 2.9E-03			
72 hr	serine-type endopeptidase inhibitor activity (5) 2.93E-03			
72 hr	structural constituent of cytoskeleton (6) 3.07E-03			
72 hr	immune response (12) 3.11E-03			
72 hr	chemotaxis (5) 3.18E-03			
72 hr	taxis (5) 3.18E-03			
72 hr	regulation of CDK activity (3) 3.46E-03			
72 hr	extracellular matrix (8) 3.51E-03			
72 hr	striated muscle thick filament (2) 3.64E-03			
72 hr	G2/M transition of mitotic cell cycle (3) 4.2E-03			
72 hr	cell motility (8) 4.21E-03			
72 hr	positive regulation of lymphocyte proliferation (2) 4.24E-03			
72 hr	regulation of T-cell proliferation (2) 4.24E-03			
72 hr	response to pest/pathogen/parasite (8) 4.87E-03			

8) Top ranked genes in IBMT, but not SMT, and vice versa for Nickel data

Table S4: Genes in top-ranked list for IBMT, but not SMT, and vice versa. For each gene, the Entrez Gene ID: Gene Symbol (Average expression level) and fold change are shown.

Time	IBMT, not SMT	SMT, not IBMT
03 hr	108014: Sfrs9 (415) -2.47	68646: 1110020G09Rik (264) -2.54
03 hr	19172: Psmb4 (810) 2.38	69697: 2310057J16Rik (155) -2.95
03 hr	69216: Ccdc23 (332) -2.81	24099: Tnfsf13b (141) -3.42
03 hr	13806: Eno1 (1174) 2.71	16671: Krt1-3 (101) -3.57
03 hr	22240: Dpysl3 (262) -3.26	AL021127: AL021127 (110) -3.54
03 hr	17120: Mad1l1 (268) -3.76	66253: Aig1 (108) -3.17
03 hr	75434: 1700001C02Rik (370) -4.23	15357: Hmgcr (128) -3.17
03 hr	234353: D430018P08 (258) -3.68	14962: H2-Bf (183) 3.53
03 hr	11857: Arhgdib (170) -4.42	16907: Lmnb2 (131) -4.01
03 hr	50724: Sap30l (235) -5.52	67235: Zfp99 (76) -4.05
03 hr	100952: Emilin1 (358) -3.62	70730: 6330409D20Rik (65) -6.45
03 hr	54614: Prpf40b (258) 3.22	72792: 2810459M11Rik (195) -3.19
03 hr	104458: Rars (291) -4.65	74356: 4931428F04Rik (99) -4.19
03 hr	19737: Rgs5 (222) -4.24	15978: Ifng (115) 3.7
03 hr	18821: Pln (142) -8.09	69698: 2310046K01Rik (91) -3.93
03 hr	59054: Mrps30 (234) -6.49	50501: Prok2 (58) -6.02
03 hr	13163: Daxx (276) -5.48	19113: Prlpe (98) -5.73
03 hr	66925: Sdhd (388) -5.12	545156: Kalrn (149) -4.17
03 hr	18113: Nnmt (107) -9.78	75799: 4930444P10Rik (45) -8.92
03 hr	72297: B3gnt3 (130) -13.21	12774: Ccr5 (58) 3.86
03 hr	78482: 1700123L14Rik (220) -15.35	16528: Kcnk4 (109) -5.51
03 hr	14767: Gpr66 (132) -13.77	71934: Car13 (67) -5.36
08 hr	72341: Tmem103 (3141) 2.22	17022: Lum (125) -2.09
08 hr	56312: Nupr1 (442) 2.15	18739: Pitpnm (165) -2.05
08 hr	68603: Pmvk (255) 2.42	70357: Kcnip1 (65) -2.13
08 hr	212706: C330016O10Rik (270) 2.25	75502: Cklfsf2b (94) -2.39
08 hr	17071: Ly6f (4703) 2.25	66275: 1810009K13Rik (189) 2.21
08 hr	15006: H2-Q1 (245) -2.23	12182: Bst1 (105) 2.29
08 hr	15433: Hoxd13 (875) 2.31	70567: 5730455O13Rik (97) -2.69
08 hr	56295: Higd1a (412) 2.74	30052: Pcsk1n (101) -2.91
08 hr	12315: Calm3 (395) -2.38	68184: Denr (138) 2.55
08 hr	18481: Pak3 (468) 2.76	80708: Pacsin3 (97) -2.56
08 hr	70235: Wdr51a (187) -2.9	73456: Izumo1 (91) 3.1
08 hr	18725: Pira2 (4812) 3.86	57249: Gabrq (110) 2.93
08 hr	16581: Kifc2 (120) -4.13	21784: Tff1 (66) -3.68
08 hr	18784: Pla2g5 (127) -5.85	18111: Nnat (94) -3.4
08 hr	12526: Cd8b (162) -4.88	74238: Mterfd3 (77) -2.95
24 hr	12409: Cbr2 (31685) -1.68	17768: Mthfd2 (65) 2.56
24 hr	30806: Adamts8 (278) 1.93	75870: Tcam1 (88) -2.14
24 hr	69202: 2610009E16Rik (6090) -1.96	14682: Gnaq (125) 2.14
24 hr	14828: Hspa5 (1442) 2.17	13835: Epha1 (143) -2.05
24 hr	14728: Gp49b (356) 2.01	74281: Spatc1 (119) -2.17
24 hr	15124: Hba-ps3 (26563) -2.26	236733: Usp11 (105) -2.17

24 hr	12051: Bcl3 (262) 2.04	74536: 9030409C19Rik (136) -2.65
24 hr	14601: Ghrh (228) 2.06	113862: V1rc5 (70) -2.78
24 hr	15526: Hspa9a (456) 2.2	70603: Mutyh (76) -3.51
24 hr	15511: Hspa1b (371) 2.38	18196: Nsg1 (66) -2.41
24 hr	27280: Phlda3 (241) 2.54	69382: 1700024P04Rik (96) -2.71
24 hr	83490: Pik3ap1 (210) -3.21	67981: Hormad1 (64) -3.72
24 hr	16740: L1Md-Tf9 (8818) 3.39	14029: Evx2 (51) -2.98
24 hr	19217: Ptger2 (177) 3.13	15061: H28 (51) -3.63
48 hr	67938: Mylc2b (4966) -2.03	56873: Lmbr1 (173) 2.4
48 hr	66734: Map1lc3a (1212) -2.18	20017: Rpo1-2 (131) 2.63
48 hr	17975: Ncl (1064) 2.23	14786: Grb7 (173) -2.62
48 hr	11830: Aqp5 (1509) -2.3	22608: Nsep1 (137) -3.03
48 hr	51938: Ccdc39 (321) -2.34	74561: Nkx6-3 (140) -2.85
48 hr	83553: Tktl1 (6955) -2.33	71355: Col24a1 (118) -3.24
48 hr	210992: Aytl2 (1476) -2.72	Y07611: Y07611 (115) -2.98
48 hr	67648: 4930542C12Rik (785) -2.63	72181: Nsun4 (65) -6.06
48 hr	110956: D17H6S56E-5 (265) 2.62	69315: 1700001L19Rik (119) -3.09
48 hr	16737: L1Md-Tf5 (14763) 2.34	80721: Slc19a3 (110) -3.9
48 hr	70223: Nars (330) 2.96	18793: Plaur (82) -2.51
48 hr	224824: Pex6 (369) -2.53	226016: 5730446C15Rik (173) -3.35
48 hr	56430: Rsn (240) 2.59	67430: 4921536K21Rik (90) -2.87
48 hr	14118: Fbn1 (502) 2.67	78548: 5430417C01Rik (84) -2.93
48 hr	12628: Cfh (993) 3.33	77080: 9230110F15Rik (112) -4.11
48 hr	13204: Dhx15 (269) 2.61	11828: Aqp3 (168) 2.99
48 hr	20763: Sprr2i (380) 2.86	14465: Gata6 (162) 2.69
48 hr	16010: Igfbp4 (772) 3.96	13003: Cspg2 (136) 3.38
48 hr	72240: 1600014C23Rik (535) -3.5	57738: Slc15a2 (88) -3.97
48 hr	19273: Ptprl (268) -3.05	100978: Nfxl1 (76) 5.06
48 hr	13587: Rnase2 (357) -2.84	74437: 4933402E13Rik (73) -4.83
48 hr	18730: Pira7 (3705) 4.63	13840: Epha6 (123) 2.8
48 hr	50926: Hnrpdl (463) 2.68	16822: Lcp2 (79) 3.57
48 hr	17912: Myo1b (219) -3.01	22222: Ubr1 (164) 3.21
48 hr	18597: Pdha1 (345) 3.13	74227: 1700016A09Rik (87) -5.79
48 hr	12331: Cap1 (755) 3.66	73456: Izumo1 (91) 7.7
48 hr	68311: Lypd2 (346) -4.22	16703: Krtap8-1 (96) -5.59
48 hr	22630: Ywhaq (600) 3.61	113862: V1rc5 (70) -3
48 hr	13711: Elf5 (168) -14.15	14126: Ms4a2 (73) -6.74
48 hr	12425: Cckar (142) -12.01	AK017085: AK017085 (89) -3.34
72 hr	69202: 2610009E16Rik (6090) -2.4	52118: Pvr (176) 2.46
72 hr	78185: 4930524L23Rik (2647) 2.57	27273: Pdk4 (137) 2.74
72 hr	15213: Hey1 (344) -2.64	14682: Gnaq (125) 2.59
72 hr	15124: Hba-ps3 (26563) -2.75	77669: 9130221D24Rik (81) 4.47
72 hr	AJ400878: AJ400878 (480) -3.04	13983: Esr2 (83) -3.08
72 hr	72461: Prcp (497) 5.36	56353: Rybp (84) 3.16
72 hr	14173: Fgf2 (206) 5.6	29820: Tnfrsf19 (89) -2.78
72 hr	12322: Camk2a (187) 7.13	78767: 2610021K21Rik (59) 9.63

9) Variance-Intensity relationship for latin-square experiment

Figure S4: The HG-U133 latin-square experiment illustrates a typical relationship between log-variance and average log-intensity after preprocessing the raw data with RMA.

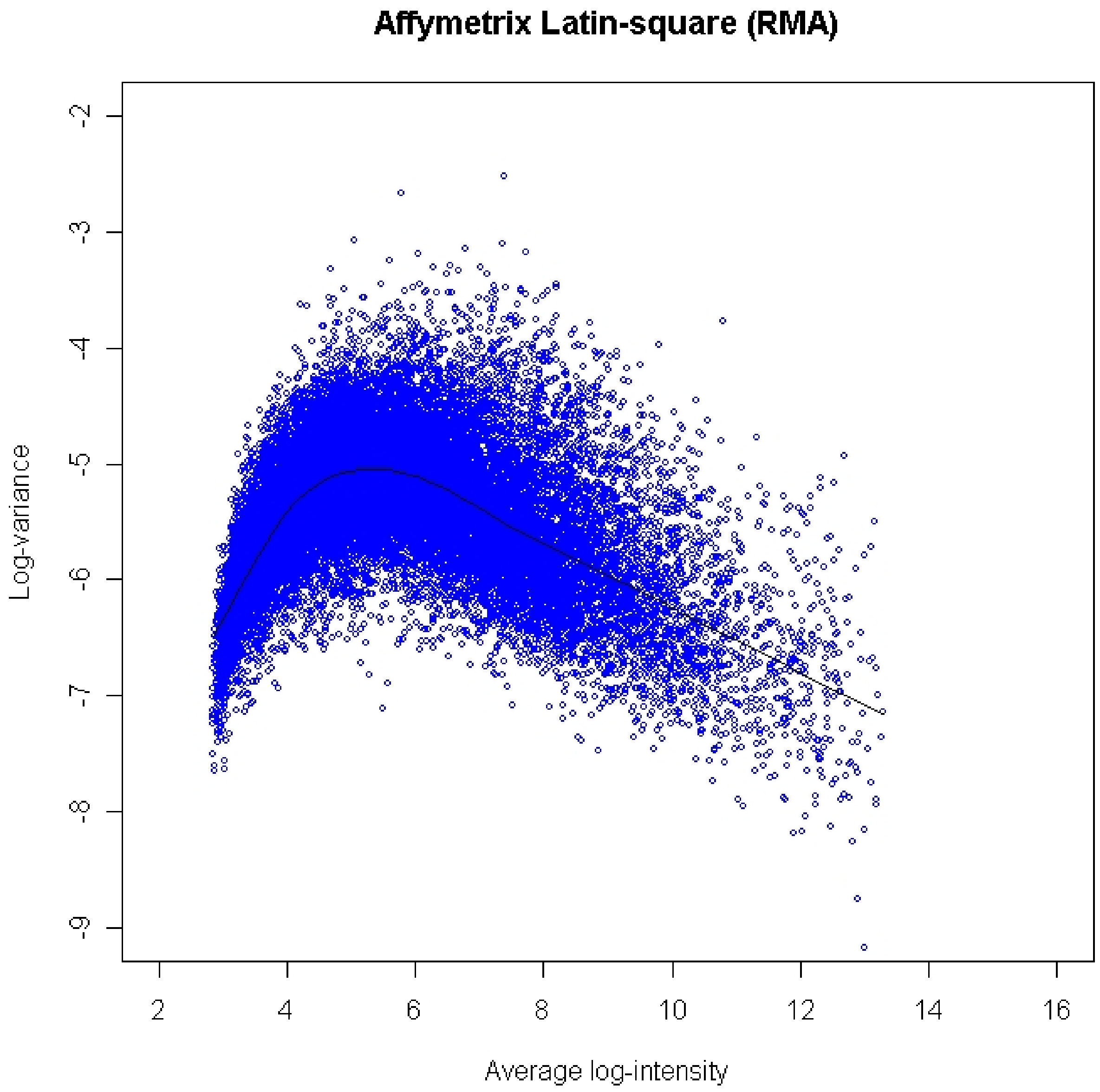

10) Robustness of method to *loess* span parameter, from latin-square experiment

Figure S5: Accumulation of false positives by gene rank using span parameters 0.1, 0.3, and 0.5 in the local regression for IBMT. Results illustrate the method's robustness to this value. Additionally, the correlations among p-values using these span values were all > 0.9995.

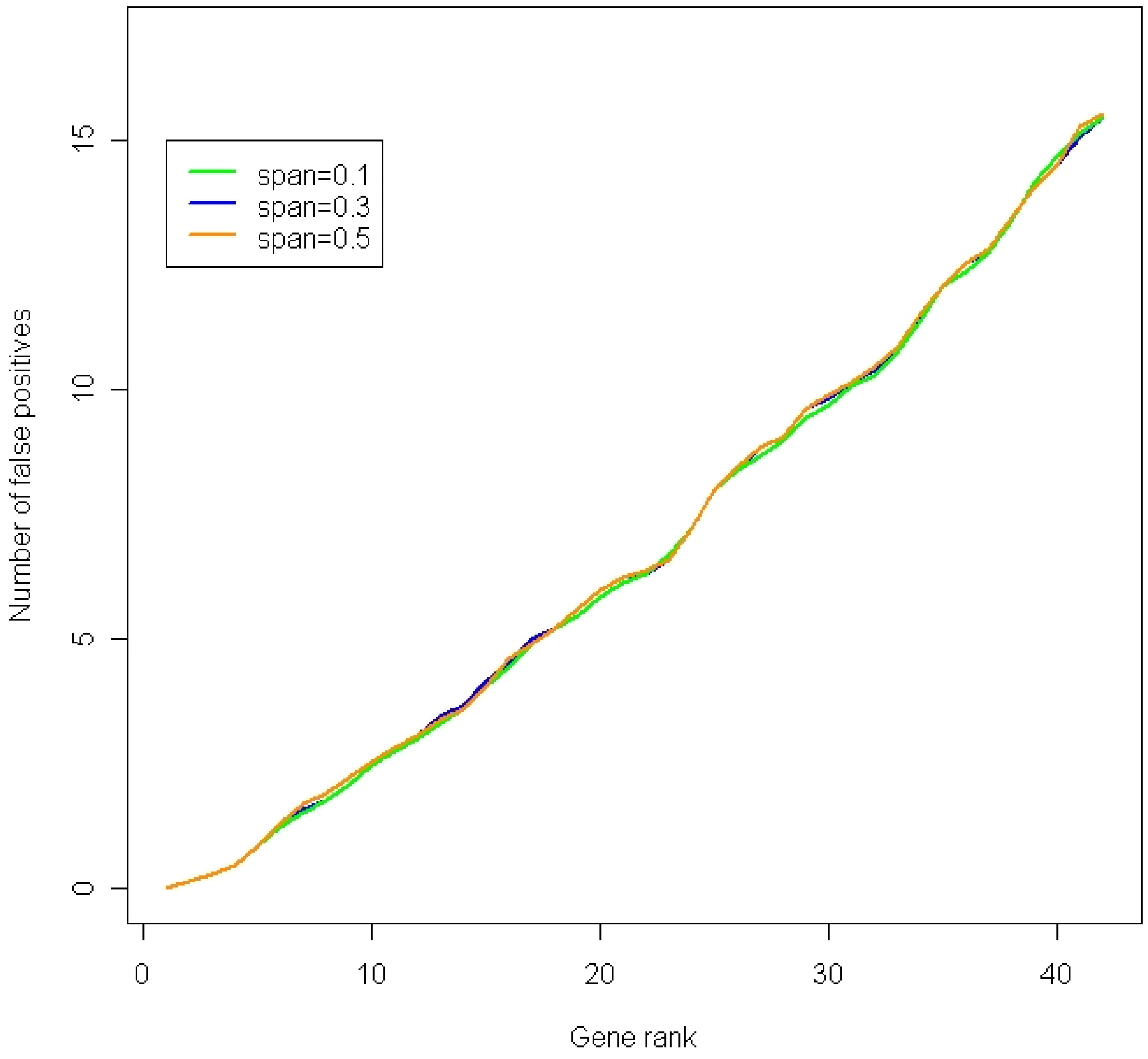

Supplement: Additional file 1 — Supplemental Material. PDF file containing several additional figures and tables. [file 1471-2105-7-538-S1.pdf]
